# Tear miRNA expression analysis reveals miR-203 as a potential regulator of corneal epithelial cells

**DOI:** 10.1186/s12886-021-02141-9

**Published:** 2021-10-25

**Authors:** Ayumi Nakagawa, Takeshi Nakajima, Mitsuyoshi Azuma

**Affiliations:** 1 Central Research Laboratories, Research and Development Division, Senju Pharmaceutical Co., Ltd., 6-4-3, Minatojima-Minamimachi, Chuo-Ku, Kobe, Hyogo 650-0047 Japan; 2 Senju Laboratory of Ocular Sciences, Senju Pharmaceutical Co., Ltd., 6-4-3, Minatojima-Minamimachi, Chuo-Ku, Kobe, Hyogo 650-0047 Japan

**Keywords:** microRNA, Tear, Microarray, Corneal epithelial cell, Viability

## Abstract

**Background:**

microRNAs (miRNAs) are small noncoding RNAs that negatively regulate gene expression. They are found within cells and in body fluids. Extracellular miRNAs have been shown to associate with the surrounding tissues. Therefore, we predicted that miRNAs in tears may contribute to regulate corneal epithelial cell function. However, information on the miRNA expression profile of tears is limited and the specific functions of tear miRNAs for corneal epithelial cells are still unknown. To study the role of tear miRNAs, we determined which miRNAs are highly expressed in tears and examined the involvement of miRNAs in corneal epithelial cell viability.

**Methods:**

miRNAs extracted from monkey tears and sera were subjected to microarray analysis. miRNAs of which expression levels were higher in tears than in sera were selected, and their expression levels were quantified by quantitative polymerase chain reaction (qPCR). To examine miRNA function, mimics and inhibitors of miRNAs were transfected into human corneal epithelial (HCE-T) cells and incubated for 24 or 48 h. After transfection of miRNA mimics and inhibitors, the viability of HCE-T cells was measured using the water soluble tetrazolium salt (WST) assay, and microarray analysis and qPCR were performed using total RNA extracted from HCE-T cells. siRNAs of the candidate targets for miR-203 were transfected into HCE-T cells and the WST assay was performed. To determine a direct target gene for miR-203, a dual luciferase reporter assay was performed in HCE-T cells using a luciferase reporter plasmid containing 3′-UTR of human IGFBP5.

**Results:**

Microarray and qPCR analyses showed that miR-184 and miR-203 were expressed significantly more highly in tears than in sera (165,542.8- and 567.8-fold, respectively, *p* < 0.05). Of these two miRNAs, transfection of a miR-203 mimic significantly reduced the viability of HCE-T cells (*p* < 0.05), while a miR-203 inhibitor significantly increased this viability (*p* < 0.05). miR-203 mimic downregulated insulin-like growth factor-binding protein 5 (IGFBP5) and nuclear casein kinase and cyclin-dependent kinase substrate 1 (NUCKS1), while miR-203 inhibitor upregulated these two genes. Transfection of IGFBP5-siRNA decreased the viability of HCE-T cells. miR-203 mimic significantly diminished the luciferase reporter activity.

**Conclusions:**

In this study, we identified miRNAs that are highly expressed in tears, and the inhibition of miR-203 increases the viability of corneal epithelial cells. Our results suggest that miR-203 contributes to regulating the homeostasis of corneal epithelial cells.

**Supplementary Information:**

The online version contains supplementary material available at 10.1186/s12886-021-02141-9.

## Background

The cornea, the anteriormost layer of the eye, is a transparent tissue and mechanical barrier that limits the entry of exogenous substances into the eye and protects the ocular tissues [1]. Based on its transparent structure, the cornea is the only part of the human body that has no blood supply; it receives nutrition through tears and aqueous humor, as well as oxygen from the air. The corneal epithelium, which is the outermost layer of the cornea, is replaced approximately every 2 weeks, and corneal wound healing is a highly regulated process that requires the proliferation and migration of epithelial cells. In tears, various proteins such as growth factors are secreted by the lacrimal gland and distributed over the cornea, where they are involved in cellular proliferation, migration, and survival. However, various chemical, physical, and pathological factors injure the corneal epithelium, resulting in corneal epithelium disorder. It is clinically important to develop a new method for treating corneal epithelium disorder.

MicroRNAs (miRNAs) are small noncoding RNAs (~ 22 nucleotides) that regulate gene expression at the post-transcriptional level by binding to the 3′-untranslated region (3′-UTR) of target messenger RNAs (mRNAs) for translational repression or degradation [2, 3]. The interaction between miRNAs and their target mRNAs requires only partial base-pairing of miRNAs, occurring at a site called the seed region (nucleotides 2-8), which allows miRNAs to target multiple genes [4]. Several studies have revealed that miRNAs play important regulatory roles in diverse cellular pathways including differentiation, organogenesis, cell proliferation, and apoptosis [5–7]. In addition, miRNAs have been reported to contribute to human diseases including ocular diseases such as glaucoma, cataract, and diabetic retinopathy [8–10].

Recently, some of the functional miRNAs previously identified in cells and tissues were found in extracellular fluids such as plasma, serum, saliva, and urine, and also in tears [11–17]. Although these studies showed the expression profile of miRNAs in patients such as Alzheimer’s disease, cancer, Sjögren syndrome, diabetic retinopathy and glaucoma, there is little information available on miRNA which is constitutively and specifically expressed in tears. miRNAs can be packaged in membrane vesicles such as exosome, released into various extracellular fluids, and transferred into cells to participate in cell-to-cell communication [16, 18, 19]. The delivered miRNAs can regulate other cells in new locations [19, 20]. Thus, we hypothesized that miRNAs present in tears might function and play important roles in maintaining the homeostasis of the ocular surface like growth factors. However, many of the available miRNA expression profiles in tears are limited in a particular disease condition and the functions of miRNAs detected in tears are not clearly understood.

The first aim of this study was to identify the dominant miRNAs in tears. We then investigated the contribution of miRNAs highly expressed in tears to the viability of corneal epithelial cells, and explored its target genes.

## Methods

### Sample collection

Tear and serum samples were collected from 4 male and 4 female cynomolgus monkeys (*Macaca fascicularis*). All of the samples were individually obtained from EveBioscience Co., Ltd. Fifty microliter of saline (Otsuka Pharmaceutical, Japan) was instilled in the nasal side of one eye by micropipette, and after a few blinking, diluted tears were collected from the temporal side. Diluted tears were repeatedly collected after an interval until sufficient volume (approximately 600 μL) was acquired. Sera were separated from approximately 2 mL blood sample drawn from the femoral vein. Animal care and all experimental procedures were performed in accordance with the guidelines of the Association for Research in Vision and Ophthalmology (ARVO) Statement for the Use of Animals in Ophthalmic and Vision Research. The protocol was approved by the Animal Welfare Committee of Research Laboratories at EveBioscience Co., Ltd.

### Microarray analysis of miRNAs

Three hundred microliter of tears and sera were centrifuged for the removal of cellular components, and their aqueous phases were gently collected. Total RNAs were extracted from each sample in accordance with the manufacturer’s protocol. The quality and quantity of total RNAs were confirmed by an Agilent 2100 Bioanalyzer (Agilent Technologies Inc., Santa Clara, CA) with an RNA 6000 Pico kit. The miRNAs extracted from monkey tears and sera were labeled with Hy5 using the miRCURY LNA Array miR labeling kit (Exiqon, Vedbaek, Denmark) and hybridized onto 3D-Gene Human miRNA Oligo chip V16.1.0.0 (Toray Industries, Tokyo, Japan), on which 1212 oligonucleotides are spotted. Hybridization signals were scanned using a 3D-Gene Scanner (Toray Industries). The raw data above a level corresponding to the average of the background plus two standard deviations were selected, and selected miRNAs were processed by background subtraction and global normalization. The miRNA profile of tears was compared with that of sera (*n* = 2 for each of tear and serum). The miRNAs which were detected in two samples were included in the analysis.

### Quantitative real-time PCR of miRNAs

Total RNAs were extracted from 4 ~ 6 samples for each of tears and sera (300 μL) using QIAzol (QIAGEN, Hilden, Germany) and miRNeasy Serum/Plasma Kit (Qiagen). To normalize for technical variation, 0.01 fmol of synthetic cel-miR-39-3p (QIAGEN) was spiked into each tear and serum sample after addition of denaturing solution. Total RNAs were treated with SUPERase-In (Thermo Fisher Scientific, Waltham, MA), and the quality and quantity of the extracted miRNAs were assessed using a spectrophotometer (NanoVue™; GE Healthcare, Little Chalfont, UK).

Four miRNAs, expressed at high levels in microarrays, were measured by quantitative polymerase chain reaction (qPCR) following the manufacturer’s instructions. Briefly, 1.12 μL of template RNAs were reverse-transcribed using miRCURY LNA RT Kit (QIAGEN), then real-time PCR was performed using miRCURY LNA SYBR Green PCR Kit (QIAGEN). The following primers were used: hsa_miR-184 miRCURY LNA miRNA PCR Assay, hsa_miR-3616-3p miRCURY LNA miRNA PCR Assay, hsa_miR-3610 miRCURY LNA miRNA PCR Assay, hsa_miR-203a-3p miRCURY LNA miRNA PCR Assay (QIAGEN). Fluorescence was monitored on a 7500 Real Time PCR system (Thermo Fisher Scientific). Relative expression levels were normalized to cel-miR-39-3p and calculated using standard curve method.

### miRNA mimics, inhibitors and siRNAs

The synthesized double-stranded full-length miRNA (22-bp) and the fully 2′-O-methylated complementary antisense oligonucleotide (22-mer) were purchased from QIAGEN (miScript miRNA Mimic: Syn-hsa-miR-184 and Syn-hsa-miR-203a-3p, miScript miRNA Inhibitor: Anti-hsa-miR-184 and Anti-hsa-miR-203a-3p, respectively). miRNA without any homology and 2′-O-methylated antisense oligonucleotides without any homology were purchased from QIAGEN (AllStars Negative Control siRNA and miScript Inhibitor Negative Control, respectively) as negative controls. siRNAs of IGFBP5, NUCKS1 and negative control were purchased from Thermo Fisher Scientific (Silencer® Select siRNAs).

### Cell cultures and transfection

Cultured immortalized human corneal epithelial (HCE-T) cells were obtained from Dr. Kaoru Sasaki at JCHO Hoshigaoka Medical Center. The cell line was authenticated with short tandem repeat (STR) analysis. The STR results showed that the DNA of the cell line completely matched HCE-T. These cells were cultured with DMEM/F12 (Invitrogen, Carlsbad, CA) supplemented with 5% fetal bovine serum (FBS, Invitrogen), 5 μg/mL insulin (Wako, Japan), 10 ng/mL human recombinant epidermal growth factor (EGF, Invitrogen), 40 μg/mL gentamycin (Invitrogen), and 100 U/mL penicillin/streptomycin (Invitrogen) at 37 °C and 5% CO_2_. Cells were seeded at 1.0 × 10^3^ cells/well in 96-well culture plates for water-soluble tetrazolium (WST) assay, 4.0 × 10^4^ cells/well in 24-well culture plates for dual luciferase reporter assay, and 9.0 × 10^4^ cells/well in 6-well culture plates for RNA extraction in DMEM/F12 medium containing 5% FBS and 40 μg/mL gentamycin on the day before transfection. At 18 h post-plating, the culture medium was replaced with DMEM/F12 medium. Transfection of miRNA mimics/ inhibitors and siRNAs was performed according to the manufacturer’s recommendations. The miRNA mimics (20 nM) and inhibitors (50 to 100 nM) were transfected into HCE-T cells with HiPerFect Transfection Reagent (QIAGEN). The 50 nM of siRNAs were transfected with Lipofectamine® RNAiMAX (Invitrogen).

To examine the baseline expression level of miR-184 and miR-203 in HCE-T cells, total RNAs were extracted from cultured cells using QIAzol and miRNeasy Mini Kit (QIAGEN) followed by reverse transcription PCR. qPCR was performed with specific primers using RNU6B (endogenous control).

### Water-soluble tetrazolium salt (WST) assay

The cell viability was determined by WST assay [21]. Forty-eight hours after transfection, WST-8 (Cell Counting Kit-8 reagent; Dojindo Laboratories, Kumamoto, Japan) was added to each well, followed by incubation in a 37 °C, 5% CO_2_ incubator for 1 h. The absorbance was measured at 450 nm in a microplate reader (BioTek, Winooski, VT). The background (WST-8 reagent absorbance) was subtracted from the data for each well. Relative changes of miRNA mimic- or inhibitor-treated cells were calculated comparing with negative control-treated cells. All assays were performed in triplicate and repeated two or three times.

### Microarray analysis and qPCR of genes

Forty-eight hours after transfection of miR-203 mimic and inhibitor, total RNA was extracted from HCE-T cells using TRIzol® Reagent (Invitrogen) and RNeasy Mini kit (QIAGEN). Subsequently, microarray analysis was performed using Genechip (Affymetrix, Santa Clara, CA). The relative expression levels of genes after transfection of mimic or inhibitor compare with their negative control were calculated (*n* = 3).

To validate the expression levels of insulin-like growth factor binding protein 5 (*IGFBP5*), nuclear casein kinase and cyclin dependent kinase substrate 1 (*NUCKS1*) and glyceraldehyde-3-phosphate dehydrogenase (*GAPDH*) mRNAs, 300 ng of template RNAs were reverse-transcribed using PrimeScript® RT Master Mix (Perfect Real Time) Kit (Takara Bio, Shiga, Japan), and real-time PCR was performed using TaqMan™ Gene Expression Master Mix (Thermo Fisher Scientific). The assay ID of TaqMan® Gene Expression Assays used as primers were following: Hs00181213_m1 (for *IGFBP5*), Hs05054673_s1 (for *NUCKS1*) and Hs02786624_g1 (for *GAPDH*). *GAPDH* was used as an internal control. Relative expression levels were calculated using standard curve method.

### miRNA target prediction

Potential target genes of miR-203 and the target sites on their 3′-UTR were predicted by miRDB (http://mirdb.org/), an online database for miRNA target prediction [22].

### Dual luciferase reporter assay

Fifty nanogram of luciferase reporter plasmid containing 3′-UTR of human IGFBP5 (HmiT100927-MT06, Genecopoeia, Rockville, MD) or pEZX-MT06 control plasmid (CmiT000001-MT06, Genecopoeia) were co-transfected with 50 nM of miR-203 mimic or negative control into HCE-T cells by Lipofectamine 2000 (Invitrogen) in 24-well culture plates. At 48 h after incubation, the firefly and *Renilla* luciferase activities were measured using the Luc-Pair™ Duo-Luciferase Assay kit 2.0 (Genecopoeia). Relative luciferase activities were calculated by normalization of firefly luciferase activities based on those of *Renilla* luciferase.

### Statistical analysis

All data of qPCR, WST assays and dual luciferase reporter assay are expressed as mean ± standard deviation (SD). Differences in these assays between groups were assessed using Student’s *t*-test. A *p* value of < 0.05 was considered statistically significant.

## Results

### miRNA expressions in tears and sera

**Fig. 1:**
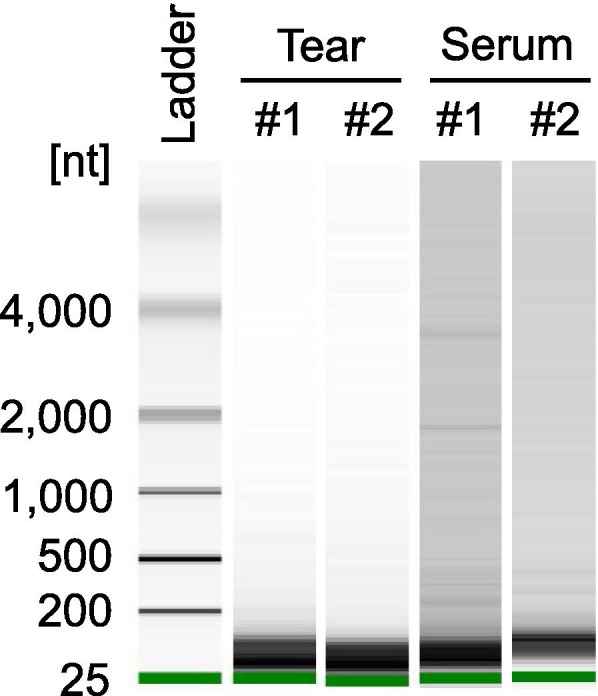
Quality validation of purified total RNAs from teas and sera. The quality of purified total RNAs obtained from tears and sera was validated using an Agilent 2100 Bioanalyzer. The gel image shows that only RNAs shorter than 200 nt were contained in each fluid (*n* = 2)

High-quality total RNAs from tears and sera were confirmed with the bioanalyzer (Fig. 1). There were only RNAs shorter than 200 nt and no ribosomal RNA, suggesting that the purified total RNAs did not contain RNAs derived from cells but contained RNAs circulating in fluids. The yields of total RNAs were 4 to 18 ng.

Microarray analysis detected 483 and 492 miRNAs in monkey tears and sera, respectively (Additional file 2). Of these, 367 were confirmed to be expressed in both tears and sera. Among these 367 miRNAs, 314 exhibited expression that was at least twice as high in tears as that in sera. Table 1 shows top miRNAs with higher expression in tears than in sera among these 314 miRNAs. On the other hand, the top miRNAs expressed higher in tears were shown in Table 2. There were few differences in the expression level between tears and sera (Additional file 2).

**Table 1:** Top 5 miRNAs with higher expression in tears than in sera

miRNA name	Averages of intensities (individual value)		Fold change (Tears/ Sera)
	Tears	Sera	
miR-184	1616.7 (1173.4, 2060.0)	19.3 (21.6, 17.1)	83.7
miR-3616-3p	1123.3 (775.1, 1471.6)	31.5 (40.1, 23.0)	35.6
miR-720^a^	7077.6 (3950.5, 10,204.6)	212.3 (262.3, 162.3)	33.3
miR-3610	471.3 (562.2, 380.4)	15.7 (16.1, 15.3)	30.1
miR-203	587.3 (184.4, 990.1)	20.4 (22.1, 18.7)	28.8

^a^The sequence annotated as miR-720 is likely to be a fragment of a transfer RNA, and it has been removed from the miRBase (https://www.mirbase.org/)

**Table 2:** Top 5 miRNAs with the higher expression in tears

miRNA name	Averages of intensities (individual value)
miR-2861	22,888.4 (14,951.8, 30,825.0)
miR-4294	22,274.8 (4206.8, 40,342.8)
miR-1908	20,012.9 (15,340.5, 24,685.4)
miR-3665	18,755.7 (15,602.1, 21,909.3)
miR-762	18,247.9 (13,612.1, 22,883.6)

### Validation of microarray analysis by qPCR

**Fig. 2:**
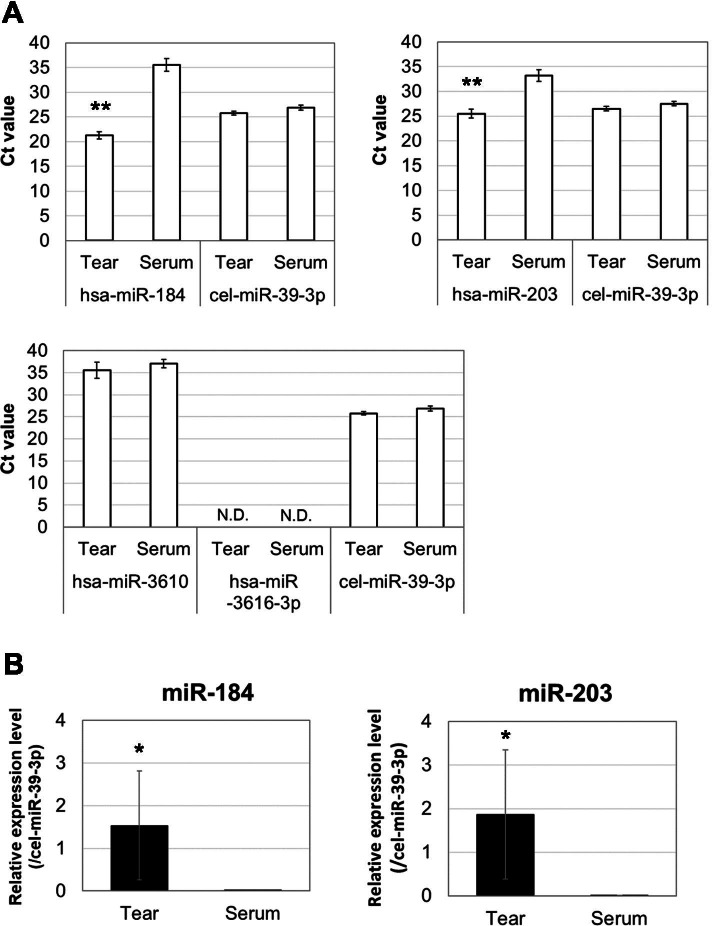
qPCR analysis of miRNAs differentially expressed between tears and sera. The average threshold cycles (Ct values) were obtained for miR-184, miR-203, miR-3610 and cel-miR-39-3p (spike-in control) (**A**). There was no significant difference in Ct value of miR-3610 between tears and sera. miR-3616-3p was not detected by qPCR in both tears and sera. miR-184 and miR-203 showed significantly higher expression in tears than in sera (**B**). Data are mean ± SD (*n* = 4 ~ 6). **p < 0.01, *p < 0.05 relative to sera (two-sided Student’s *t*-test)

miR-720, which is likely to be a fragment of a transfer RNA, has now been removed from the miRNA database (miRBase; https://www.mirbase.org/). Therefore, the expression levels of the top 4 miRNAs, miR-184, miR-3616-3p, miR-3610, and miR-203, were validated by qPCR. The average threshold cycles (Ct values) of miR-184 and miR-203 in tears were 21.3 and 25.5, respectively, and those in sera were 35.6 and 33.2, respectively (Fig. 2A). The Ct value of miR-3610 was higher than 35 in both tears and sera, and there was no significant difference between them. miR-3616-3p was not detected by qPCR in both tears and sera. Two of the four miRNAs, miR-184 and miR-203, showed significantly higher expression in tears than in sera (Fig. 2B, 165,542.8- and 567.8-fold, respectively, *p* < 0.05). Thus, two dominant miRNAs in tears, miR-184 and miR-203, were selected to identify their effects on HCE-T cells.

### Contribution of miRNA to the viability of HCE-T cells

**Fig. 3:**
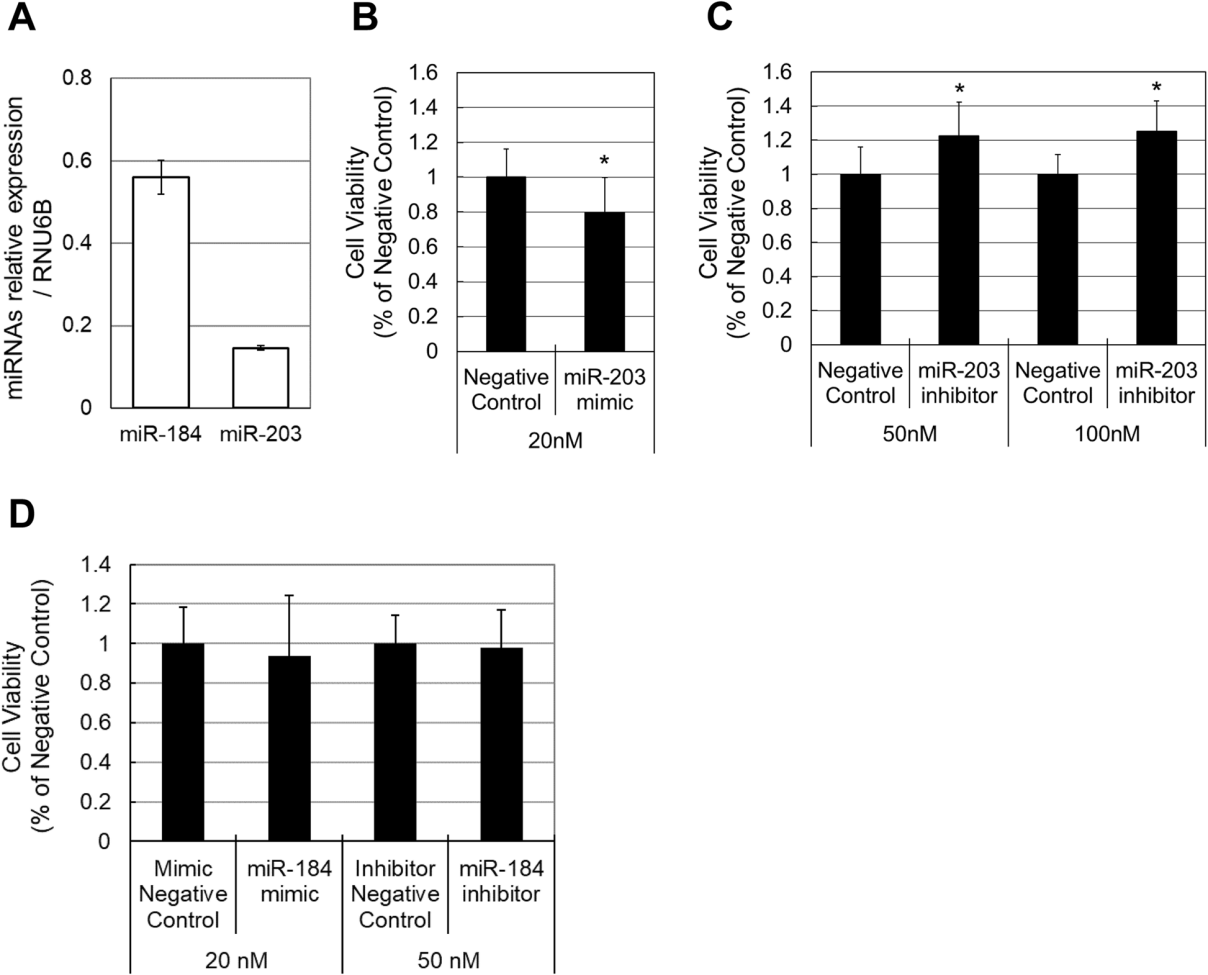
Contribution of miR-203 and miR-184 to the viability of corneal epithelial cells. The baseline expression level of miRNAs (**A**). The viability of HCE-T cells was significantly decreased by the miR-203 mimic (**B**) and was significantly increased by the miR-203 inhibitor (**C**). The viability of HCE-T cells was not changed by the mimic and inhibitor of miR-184 (**D**). Data are mean ± SD (*n* = 6 for miR-203 mimic, *n* = 9 for miR-203 inhibitor, and *n* = 9 for mimic and inhibitor of miR-184). *p < 0.05 relative to the negative control (two-sided Student’s *t*-test)

We next studied the contributions of the two relatively highly expressed miRNAs in tears, miR-184 and miR-203, to regulating the viability of corneal epithelial cells. The baseline expression levels of miR-184 and miR-203 compared with that of RNU6B (endogenous control) were 0.56 and 0.15, respectively (Fig. 3A). The cell viability was significantly decreased to 0.8-fold by the miR-203 mimic (*p* < 0.05) and was significantly enhanced 1.2-fold by miR-203 inhibitor (*p* < 0.05) comparing with these of the negative controls (Fig. 3B and C). In contrast, mimic and inhibitor of miR-184 did not change the cell viability (Fig. 3D).

### Transcripts in HCE-T cells changed by miR-203 mimic and inhibitor

To identify target mRNAs for miR-203 in HCE-T cells, we confirmed the identity of the transcripts altered after transfection of miR-203 mimic and inhibitor in HCE-T cells by microarray analysis (Table 3). At 48 h after transfection, seven genes, IGFBP5, GNAO1, CDKN2B, NUCKS1, FRAS1, TLL1, and ARID5B, were downregulated by miR-203 mimic and upregulated by miR-203 inhibitor (cut-off: > 1.3-fold). In contrast, three genes AREG, PSTPIP2, and NFATC2, were upregulated by miR-203 mimic and downregulated by miR-203 inhibitor (cut-off: > 1.3-fold).

**Table 3:** Gene transcripts changed by miR-203 mimic and inhibitor

Gene symbol	Intensity		Fold change (/ NC)	Intensity		Fold change (/ NC)
	miR-203 Mimic	Mimic NC		miR-203 Inhibitor	Inhibitor NC	
IGFBP5	36.76	63.56	−1.72	80.45	48.50	1.66
GNAO1	38.85	72.00	−1.85	67.18	46.85	1.44
CDKN2B	58.08	95.01	−1.64	105.42	68.59	1.54
NUCKS1	48.84	83.29	−1.7	98.36	72.50	1.36
FRAS1	70.52	112.99	−1.6	126.24	90.51	1.4
TLL1	199.47	278.20	−1.39	254.23	184.82	1.37
ARID5B	120.26	160.90	−1.34	170.07	128.89	1.32
AREG	44.02	32.45	1.36	33.59	44.63	−1.33
PSTPIP2	310.83	215.27	1.45	177.29	238.86	−1.35
NFATC2	106.15	54.95	1.94	45.25	62.25	−1.38

*NC* Negative control, *IGFBP5* Insulin-like growth factor-binding protein 5, *GNAO1* G protein subunit alpha o1, *CDKN2B* Cyclin dependent kinase inhibitor 2B, NUCKS1 Nuclear casein kinase and cyclin-dependent kinase substrate 1, *FRAS1* Fraser extracellular matrix complex subunit 1, *TLL1* Tolloid like 1, *ARID5B* AT-rich interaction domain 5B, *AREG* Amphiregulin, *PSTPIP2* Proline-serine-threonine phosphatase interacting protein 2, *NFATC2* Nuclear factor of activated T cells 2

### Identification a direct target gene for miR-203

**Fig. 4:**
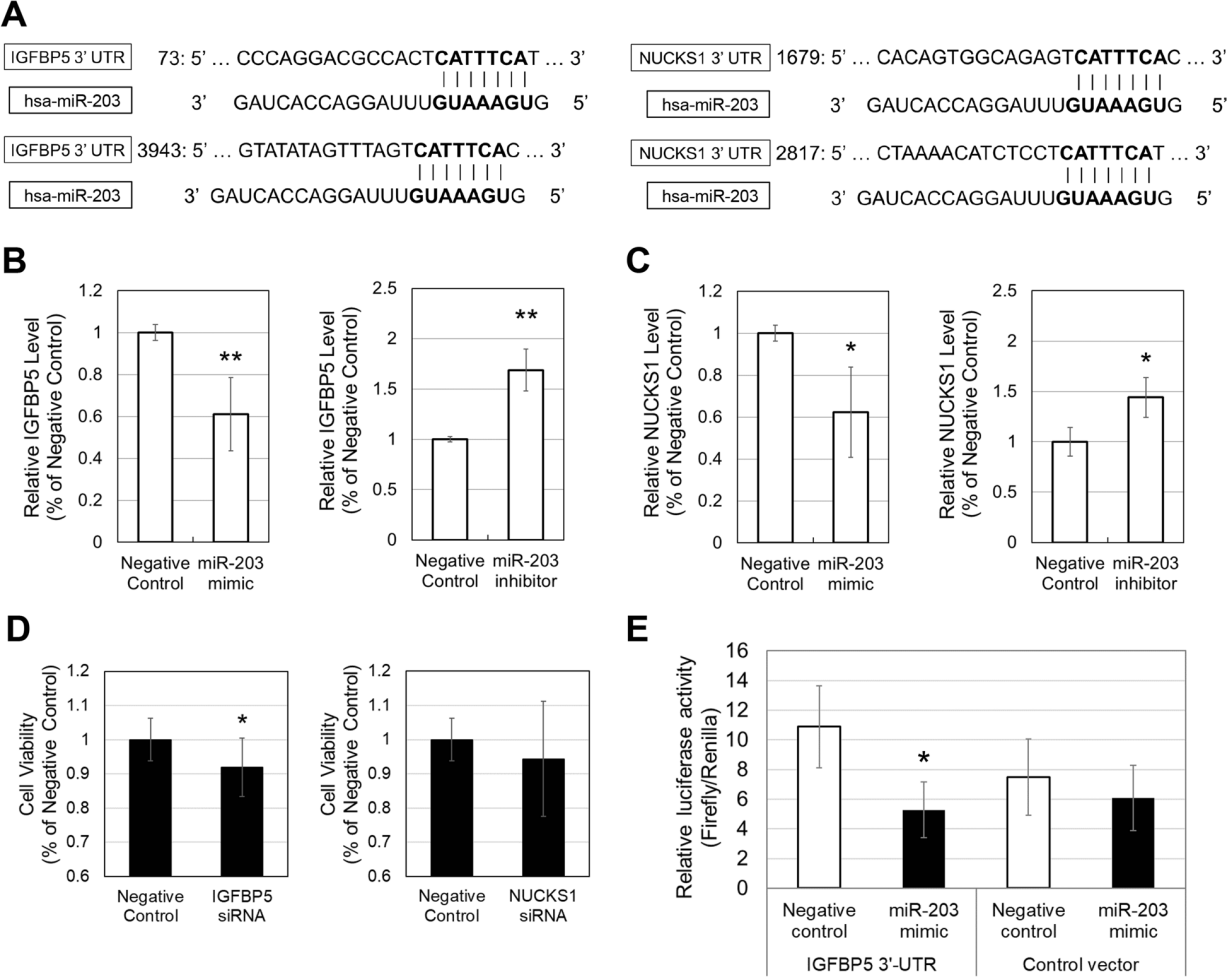
The potential target genes of miR-203. There were two putative target sites of miR-203 in the 3′-UTR of IGFBP5 and NUCKS1 mRNA (**A**). qPCR analysis revealed that the relative expression levels of IGFBP5 (**B**) and NUCKS1 (**C**) were significantly decreased by the miR-203 mimic (20 nM) and were significantly increased by the miR-203 inhibitor (75 nM) comparing with the negative controls. Data are mean ± SD (*n* = 4). **p < 0.01, *p < 0.05 relative to the negative control (two-sided Student’s *t*-test). The viability of HCE-T cells was significantly decreased by the IGFBP5-siRNA comparing with the negative controls (**D**). NUCKS1-siRNA had no significantly effect on the cell viability. Data are mean ± SD (*n* = 6). *p < 0.05 relative to the negative control (one-sided Student’s *t*-test). The luciferase reporter activity using a luciferase reporter plasmid containing 3′-UTR of human IGFBP5 was significantly diminished after the transfection of miR-203 mimic (**E**). Data are mean ± SD (*n* = 3). *p < 0.05 relative to the negative control (one-sided Student’s *t*-test)

To screen the target genes of miR-203 among 10 genes which was identified by microarray analysis, miR-203 target sites were identified by an in silico miRNA target prediction search. IGFBP5 and NUCKS1 mRNA had two putative target sites in their 3′-UTR (Fig. 4A). In order to validate the change of expression level of IGFBP5 and NUCKS1 in microarray analysis, we then performed qPCR analysis for these two genes 24 h and 48 h after transfection of miR-203 mimic and inhibitor into HCE-T cells. At 24 h, the expression of IGFBP5 and NUCKS1 were significantly downregulated by the miR-203 mimic (*p* < 0.01) and were significantly upregulated by the miR-203 inhibitor (*p* < 0.01) comparing with the negative controls (Fig. 4B and C). The effects induced by miR-203 mimic and inhibitor were reduced at 48 h (data not shown).

The effect of IGFBP5 and NUCKS1 silencing on the viability of HCE-T cells was determined using siRNAs of these genes. IGFBP5-siRNA significantly decreased the cell viability of HCE-T comparing with the negative control (Fig. 4D, *p* < 0.05). In contrast, NUCKS1-siRNA had no significantly effect on the cell viability. To further determine whether IGFBP5 is a direct target gene for miR-203, a dual luciferase reporter assay was performed in HCE-T cells. The luciferase reporter activity was significantly diminished after the transfection of miR-203 mimic compared with transfection of negative control of miRNA (Fig. 4E). These results demonstrated that miR-203 negatively regulates the proliferation of HCE-T cells by targeting IGFBP5.

## Discussion

Several miRNAs have been reported to be specific to individual organs, tissues, or cell types. For example, miR-122 and miR-124 are specifically expressed in liver and brain, respectively [23–27]. In addition, Weber et al. reported that the composition and concentrations of miRNAs differ among several body fluid types [11]. These results demonstrate that specific miRNAs are generated at each cell and secreted into body fluids to act in the specific site. Therefore, we hypothesized that miRNAs express higher in tear than in other body fluid may play key roles in the homeostasis of ocular surfaces such as corneal epithelial cells.

In this study, we characterized the miRNA expression profiles of two body fluids, tears and sera. Several studies on miRNA expression in tears have been reported [12–17]. Although these studies showed the difference of miRNAs expression between patients and healthy controls, little information is currently available on miRNA which is constitutively and specifically expressed in tears. Blood circulates throughout the body and maintains the homeostasis of various organs, while tears supply oxygen and nutrition to the ocular surface and maintain its homeostasis. For this reason, sera fractionated from blood were used as a control body fluid to determine miRNAs specific for tears. Dufourd T et al. reported that there were differences of miRNA quality, content and profile between plasma and serum in rat while no differences were found between them in human because of its large blood volume. Therefore, monkey serum and plasma also might not show important differences in miRNA profile because of their comparably large blood volume similar to human [28]. We analyzed the miRNA expression profiles in tears of monkey using human probes because the majority of monkey miRNAs have been well conserved in the human genome [29]. We revealed that miR-203 expressed higher in tears than sera. The viability of HCE-T cells was significantly increased 1.2-fold by miR-203 inhibitor compared with negative control (Fig. 3). In the immortalized HCE cell line, the proliferation was promoted approximately 1.5-fold even by standard growth supplements [30], indicating the effect of miR-203 inhibitor was significant for the viability of HCE-T cells. Several studies demonstrated that the effect of the drug on the viability of HCE-T cells can be linked to the effect on the corneal wound healing [31–34]. Taken together, miR-203 inhibitor may have a therapeutic potential in corneal epithelial wound healing.

miR-203 is widely known as a tumor suppressor and skin-related miRNA, and plays important roles in cell proliferation, differentiation, and metastasis by controlling various genes [35–41]. In the skin, miR-203 represses stemness and promote epidermal differentiation by restricting proliferative potential [36, 37]. miR-203 may also control corneal differentiation by inhibiting its proliferation to balance between them and maintain its homeostasis.

Our microarray analysis and qPCR analysis revealed that miR-203 mimic downregulates IGFBP5 and NUCKS1, and miR-203 inhibitor upregulates this gene (Table 3 and Fig. 4B). In silico analysis demonstrated that miR-203 directly recognizes the 3′-UTR of the IGFBP5 and NUCKS1 mRNAs by its positions 2 to 8 (Fig. 4A). Luciferase reporter assay demonstrated that IGFBP-5 is a direct target gene for miR-203 (Fig. 4E). IGFBP5 have been reported to be involved in cell proliferation, migration and/or adhesion [42, 43]. Another study demonstrated that IGFBP5 enhances wound closure of mammary epithelial cells during injury by inhibiting the pro-fibrotic/pro-metastatic actions of transforming growth factor-β1 (TGF-β1) [44]. In this study, we also revealed that silencing of IGFBP5 resulted in decreasing the cell viability of HCE-T cells. These findings support that miR-203 directory regulates IGFBP5 expression to control the viability of corneal epithelial cells, and inhibiting miR-203 enhanced its viability. In contrast, the silencing of NUCKS1 had no significantly effect on the cell viability of HCE-T cells. Previous studies demonstrated that NUCKS1 associates with cell proliferation in tumor [45], while the silencing of NUCKS1 facilitates corneal wound healing following alkali injury [46]. Taken together, these and our results indicated that NUCKS1 may have a different effect on the cell proliferation in different cells or conditions.

miR-184, other highly expressed miRNA in tears, did not show an association with any change in the viability of HCE-T cells. However, miR-184, which showed the highest relative expression level in tears compared with that in sera, has been reported to negatively regulate corneal lymphangiogenesis and angiogenesis [47–49]. These findings indicate that miR-184 in tears may be important for maintaining corneal homeostasis to prevent inflammation and neovascularization.

A limitation of this study is that we confirmed the function of miR-203 in the steady state cell culture, not in the disease condition. Viticchie et al. reported that miR-203 was downregulated to increase the expression of its targets which are necessary for cell proliferation and migration in keratinocytes of mouse skin epidermis during injury [38]. They demonstrated that miR-203 repression could mediate the skin re-epithelialization during wound healing. Furthermore, An J et al reported that miR-203 was downregulated in mouse corneal epithelium during corneal epithelial injury [50]. Their study suggested the possibility that inhibiting of miR-203 during corneal epithelial injury might increase the viability of corneal epithelial cells as shown in this study. Further studies are needed to understand the function of miR-203 in corneal epithelial disease. Moreover, the circulating miRNA can reportedly be a biomarker for several diseases [51–55]. Thus, the specific and/or abundant miRNAs in tears might have the potential to be valuable biomarkers for ocular diseases.

## Conclusion

The abundant miRNAs in tears would play important roles to maintain the homeostasis of ocular surface. In particular, our study provides evidence that miR-203 in tears is an important factor in controlling the physiology of corneal epithelial cells. The effect of inhibiting miR-203 on enhancing corneal epithelial cell viability supports its potential as a therapeutic role in corneal epithelial wound healing.

## Supplementary Information

**Additional file 1.** The gel images of capillary electrophoresis for total RNAs from teas and sera. This data is the uncropped gel images of capillary electrophoresis for total RNAs from teas and sera using the Agilent 2100 bioanalyzer. Microarray analysis was performed for each 2 sample of tear and serum (#1 and #2 in this figure).

**Additional file 2.** The intensities of microarray analysis for individual miRNAs in monkey tears and sera. This data is the full result table of the microarray analysis of miRNA in tears and sera.

**Additional file 3.** The viability of HCE-T cells after transfection with mimic or inhibitor of miR-203 and miR-184. This data is the raw data of WST assay of HCE-T cells after transfection with miR-203 mimic (A, 2 experiments, triplicate), miR-203 inhibitor (B, 3 experiments, triplicate), and mimic and inhibitor of miR-184 (C, 3 experiments, triplicate).

## Data Availability

The datasets during and/or analyzed during the current study are available from the corresponding author on reasonable request.

## Abbreviations

miRNA: microRNA
3′-UTR: 3′-untranslated region
mRNA: Messenger RNA
qPCR: Quantitative polymerase chain reaction
IGFBP5: Insulin-like growth factor binding protein 5
NUCKS1: Nuclear casein kinase and cyclin dependent kinase substrate 1
GAPDH: Glyceraldehyde-3-phosphate dehydrogenase
HCE-T: Human corneal epithelial
DMEM: Dulbecco’s minimum essential medium
FBS: Fetal bovine serum
EGF: Epidermal growth factor
WST: Water-soluble tetrazolium salt
SD: Standard deviation
GNAO1: G protein subunit alpha o1
CDKN2B: Cyclin dependent kinase inhibitor 2B
FRAS1: Fraser extracellular matrix complex subunit 1
TLL1: Tolloid like 1
ARID5B: AT-rich interaction domain 5B
AREG: Amphiregulin
PSTPIP2: Proline-serine-threonine phosphatase interacting protein 2
NFATC2: Nuclear factor of activated T cells 2
NC: Negative control
TGF-β1: Transforming growth factor-β1
