# Supplementary figures and images for: Tear miRNA expression analysis reveals miR-203 as a potential regulator of corneal epithelial cells
Source: BMC Ophthalmol. 2021 Oct 25;21:377. doi: 10.1186/s12886-021-02141-9 (PMC8543880; doi:10.1186/s12886-021-02141-9)

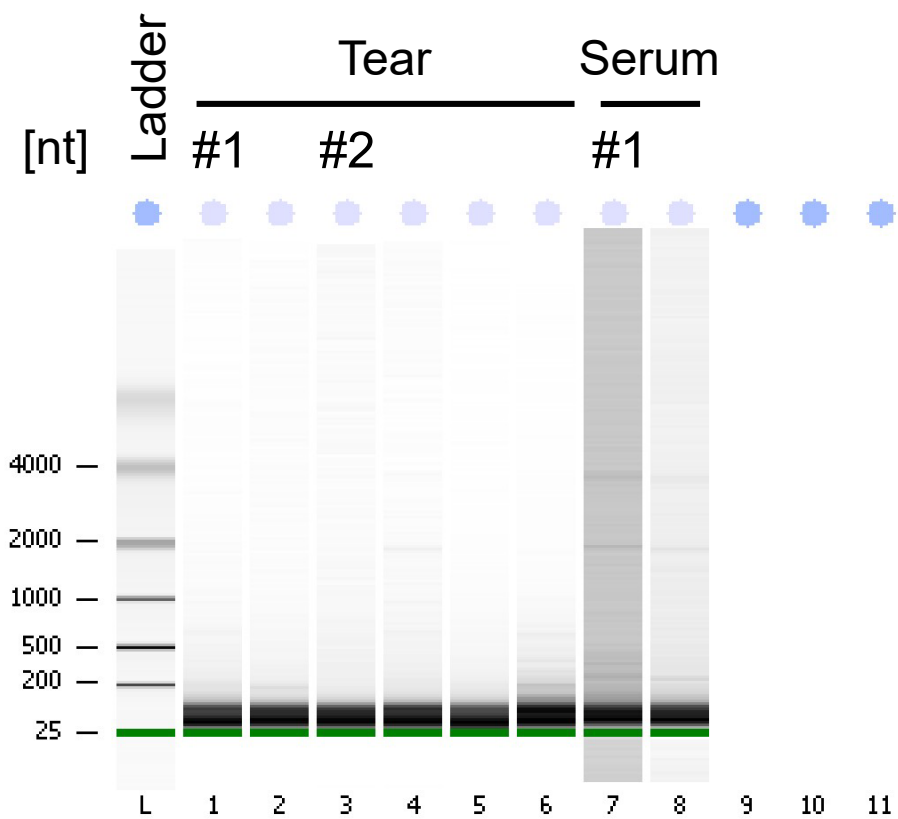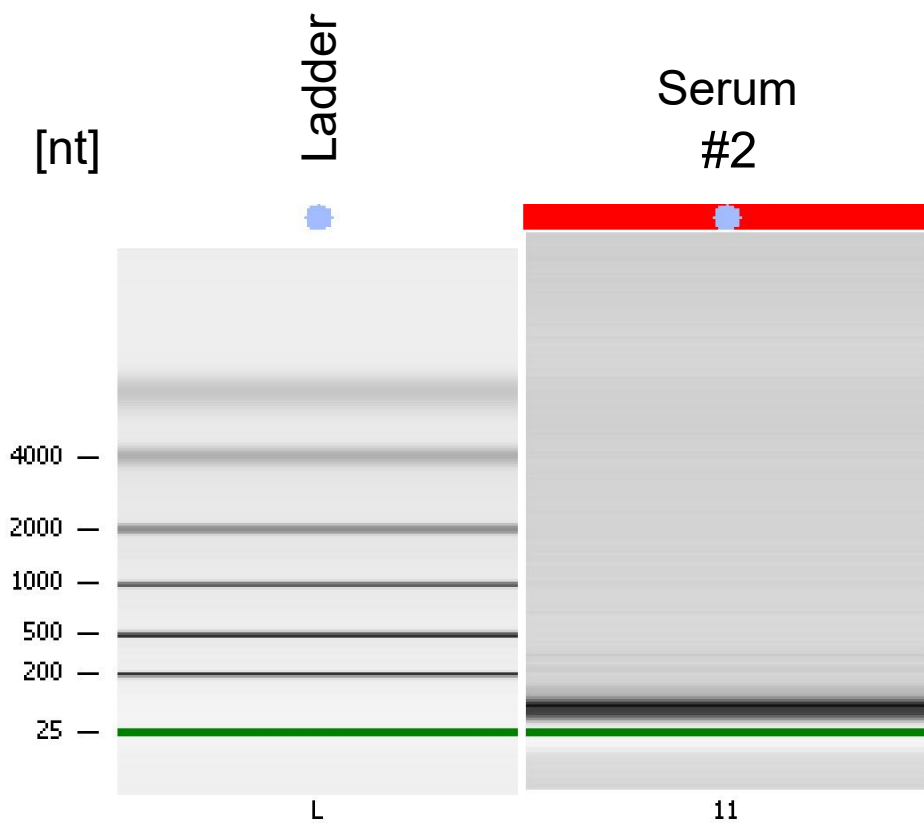

Supplement: Supplementary file 1 — Additional file 1. The gel images of capillary electrophoresis for total RNAs from teas and sera. This data is the uncropped gel images of capillary electrophoresis for total RNAs from teas and sera using the Agilent 2100 bioanalyzer. Microarray analysis was performed for each 2 sample of tear and serum (#1 and #2 in this figure). [file 12886_2021_2141_MOESM1_ESM.pdf]

**A**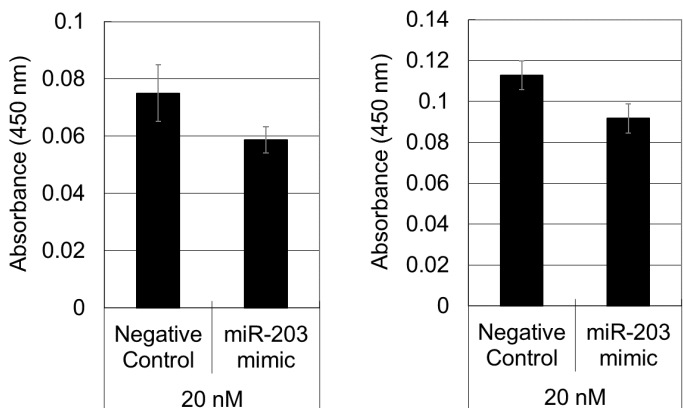**B**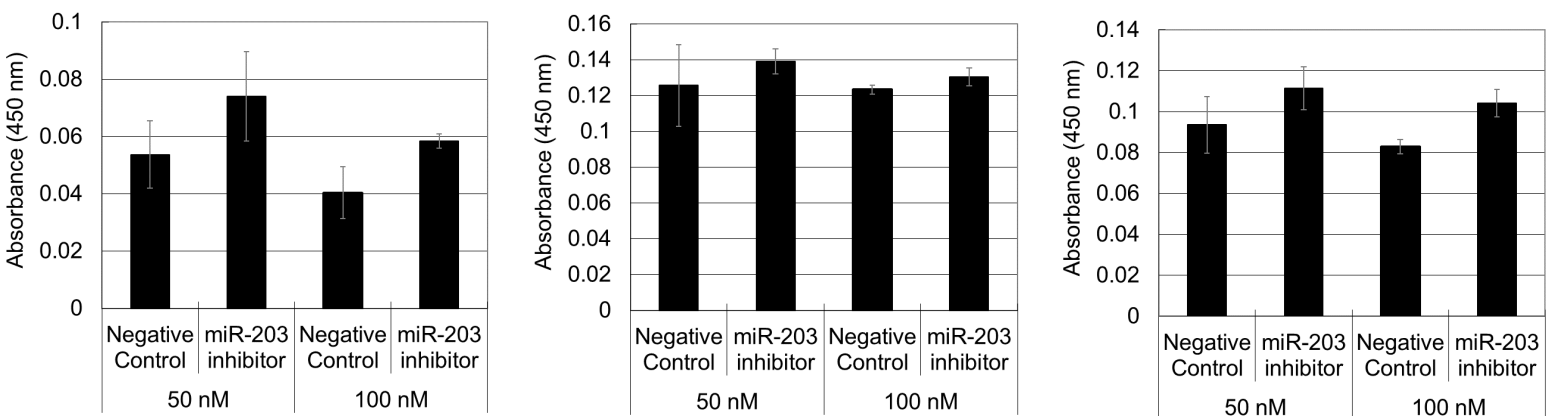**C**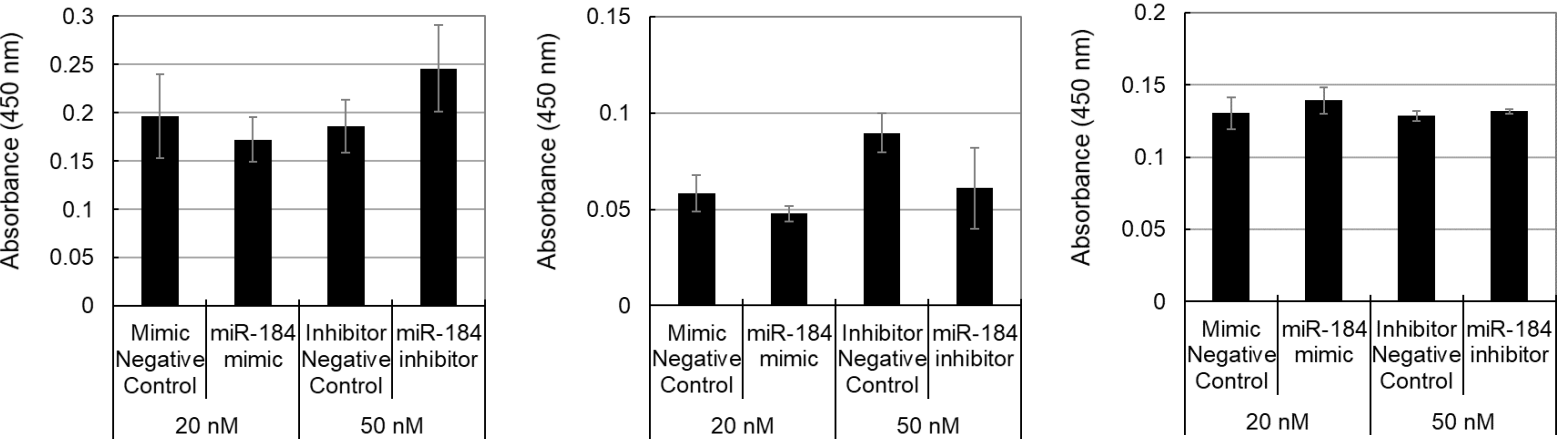

Supplement: Supplementary file 3 — Additional file 3. The viability of HCE-T cells after transfection with mimic or inhibitor of miR-203 and miR-184. This data is the raw data of WST assay of HCE-T cells after transfection with miR-203 mimic (A, 2 experiments, triplicate), miR-203 inhibitor (B, 3 experiments, triplicate), and mimic and inhibitor of miR-184 (C, 3 experiments, triplicate). [file 12886_2021_2141_MOESM3_ESM.pdf]
