# Supplementary material for: Tear miRNA expression analysis reveals miR-203 as a potential regulator of corneal epithelial cells
Source: BMC Ophthalmol. 2021 Oct 25;21:377. doi: 10.1186/s12886-021-02141-9 (PMC8543880; doi:10.1186/s12886-021-02141-9)
Supplement: Supplementary file 2 — Additional file 2. The intensities of microarray analysis for individual miRNAs in monkey tears and sera. This data is the full result table of the microarray analysis of miRNA in tears and sera. [file 12886_2021_2141_MOESM2_ESM.pdf]

Name	ID	Normalized		Average	SD	Normalized		Average	SD
		Tear #1	Tear #2			Serum #1	Serum #2		
hsa-let-7a-2*	MIMAT0010195	54.3	376.7	215.5	227.9	38.3	24.1	31.2	10.1
hsa-let-7b	MIMAT0000063	55.1	628.6	341.9	405.5	84.1	16.3	50.2	48.0
hsa-let-7b*	MIMAT0004482	137.0	145.6	141.3	6.1	48.3	20.8	34.6	19.5
hsa-let-7d	MIMAT0000065	N.D.	901.1	-	-	52.5	17.3	34.9	24.9
hsa-let-7d*	MIMAT0004484	57.5	110.7	84.1	37.6	100.0	32.3	66.2	47.9
hsa-let-7f-1*	MIMAT0004486	116.7	108.4	112.6	5.9	14.2	20.2	17.2	4.3
hsa-let-7i	MIMAT0000415	N.D.	454.0	-	-	171.5	26.1	98.8	102.8
hsa-miR-103	MIMAT0000101	N.D.	371.4	-	-	241.4	34.9	138.1	146.0
hsa-miR-103-as	MIMAT0007402	N.D.	N.D.	-	-	11.9	13.8	12.9	1.4
hsa-miR-106a	MIMAT0000103	N.D.	194.5	-	-	521.9	50.1	286.0	333.6
hsa-miR-106b	MIMAT0000680	N.D.	205.4	-	-	512.1	82.7	297.4	303.6
hsa-miR-106b*	MIMAT0004672	59.0	62.1	60.5	2.2	32.2	24.0	28.1	5.8
hsa-miR-107	MIMAT0000104	N.D.	213.2	-	-	182.1	29.6	105.8	107.8
hsa-miR-1178	MIMAT0005823	46.0	50.8	48.4	3.4	11.2	19.8	15.5	6.0
hsa-miR-1180	MIMAT0005825	53.9	82.9	68.4	20.6	44.4	20.7	32.5	16.7
hsa-miR-1181	MIMAT0005826	195.2	198.5	196.9	2.3	88.6	53.5	71.1	24.8
hsa-miR-1182	MIMAT0005827	123.1	119.1	121.1	2.9	18.9	25.8	22.3	4.9
hsa-miR-1184	MIMAT0005829	50.5	58.0	54.2	5.3	15.0	15.6	15.3	0.4
hsa-miR-1193	MIMAT0015049	276.9	254.3	265.6	15.9	68.3	57.6	62.9	7.5
hsa-miR-1200	MIMAT0005863	54.7	91.0	72.8	25.6	36.8	18.8	27.8	12.7
hsa-miR-1202	MIMAT0005865	700.0	432.3	566.1	189.3	51.5	45.6	48.6	4.2
hsa-miR-1203	MIMAT0005866	93.7	179.6	136.7	60.8	226.2	135.0	180.6	64.5
hsa-miR-1204	MIMAT0005868	90.1	129.0	109.5	27.5	27.9	24.7	26.3	2.2
hsa-miR-1207-3p	MIMAT0005872	74.1	100.0	87.1	18.3	30.8	31.3	31.1	0.4
hsa-miR-1207-5p	MIMAT0005871	883.3	1414.9	1149.1	375.9	438.2	209.5	323.9	161.7
hsa-miR-1208	MIMAT0005873	85.6	110.4	98.0	17.5	14.5	N.D.	-	-
hsa-miR-122	MIMAT0000421	N.D.	42.4	-	-	16.9	14.4	15.6	1.8
hsa-miR-1224-3p	MIMAT0005459	225.9	380.3	303.1	109.2	93.9	56.6	75.3	26.4
hsa-miR-1224-5p	MIMAT0005458	410.7	284.1	347.4	89.5	37.8	17.8	27.8	14.1
hsa-miR-1225-3p	MIMAT0005573	165.2	140.1	152.6	17.8	53.1	41.9	47.5	7.9
hsa-miR-1225-5p	MIMAT0005572	516.4	634.5	575.5	83.5	380.2	295.7	338.0	59.8
hsa-miR-1226	MIMAT0005577	116.1	185.4	150.8	49.1	64.6	29.6	47.1	24.8
hsa-miR-1226*	MIMAT0005576	183.3	80.4	131.9	72.8	16.7	N.D.	-	-
hsa-miR-1227	MIMAT0005580	109.5	92.1	100.8	12.3	25.0	20.3	22.6	3.4
hsa-miR-1228	MIMAT0005583	410.6	463.0	436.8	37.1	177.8	112.3	145.0	46.3
hsa-miR-1228*	MIMAT0005582	5982.4	14559.1	10270.7	6064.6	12623.1	8488.8	10556.0	2923.4
hsa-miR-1229	MIMAT0005584	284.0	405.5	344.7	85.9	128.4	78.6	103.5	35.2
hsa-miR-1231	MIMAT0005586	159.8	96.6	128.2	44.7	71.0	21.7	46.3	34.8
hsa-miR-1233	MIMAT0005588	241.6	322.8	282.2	57.4	135.1	97.2	116.2	26.8
hsa-miR-1234	MIMAT0005589	229.6	241.8	235.7	8.7	42.1	28.4	35.2	9.7
hsa-miR-1236	MIMAT0005591	239.4	373.2	306.3	94.6	98.1	49.6	73.8	34.3
hsa-miR-1237	MIMAT0005592	202.8	316.7	259.8	80.6	112.6	58.9	85.7	38.0
hsa-miR-1238	MIMAT0005593	227.1	322.6	274.8	67.5	116.8	91.3	104.0	18.1
hsa-miR-124	MIMAT0000422	289.9	143.1	216.5	103.8	N.D.	N.D.	-	-
hsa-miR-1246	MIMAT0005898	3261.9	8389.7	5825.8	3625.9	379.0	131.7	255.4	174.8
hsa-miR-1247	MIMAT0005899	44.8	98.1	71.5	37.7	15.0	17.9	16.4	2.1
hsa-miR-1249	MIMAT0005901	183.7	348.9	266.3	116.8	144.5	82.9	113.7	43.6
hsa-miR-1254	MIMAT0005905	338.1	237.2	287.7	71.4	16.2	14.7	15.4	1.1
hsa-miR-125a-3p	MIMAT0004602	96.0	346.0	221.0	176.8	51.2	62.9	57.0	8.3
hsa-miR-125a-5p	MIMAT0000443	67.9	423.8	245.9	251.6	31.9	N.D.	-	-
hsa-miR-125b	MIMAT0000423	65.4	617.4	341.4	390.3	15.3	14.6	15.0	0.5
hsa-miR-125b-2*	MIMAT0004603	51.2	77.2	64.2	18.4	N.D.	20.9	-	-
hsa-miR-126	MIMAT0000445	N.D.	53.9	-	-	690.2	18.4	354.3	475.1
hsa-miR-1260	MIMAT0005911	1687.1	1413.9	1550.5	193.2	173.8	97.0	135.4	54.3

hsa-miR-1260b	MIMAT0015041	13950.2	9513.6	11731.9	3137.1	2982.5	2260.5	2621.5	510.5
hsa-miR-1266	MIMAT0005920	68.2	107.4	87.8	27.7	13.7	N.D.	-	-
hsa-miR-1268	MIMAT0005922	4835.9	7172.7	6004.3	1652.4	12023.2	6263.6	9143.4	4072.6
hsa-miR-1269	MIMAT0005923	N.D.	66.4	-	-	15.9	15.2	15.6	0.4
hsa-miR-1271	MIMAT0005796	44.2	92.0	68.1	33.8	29.4	25.2	27.3	3.0
hsa-miR-1273	MIMAT0005926	125.0	224.1	174.5	70.1	25.0	16.3	20.6	6.1
hsa-miR-1273c	MIMAT0015017	118.3	92.0	105.2	18.6	16.1	22.6	19.4	4.6
hsa-miR-1273d	MIMAT0015090	88.4	102.5	95.4	10.0	17.8	17.8	17.8	0.0
hsa-miR-1273e	MIMAT0018079	94.7	662.6	378.7	401.5	26.2	16.4	21.3	6.9
hsa-miR-1274a	MIMAT0005927	85.6	382.6	234.1	210.0	128.8	74.0	101.4	38.8
hsa-miR-1274b	MIMAT0005938	1383.8	8787.4	5085.6	5235.1	1719.5	704.0	1211.7	718.0
hsa-miR-1275	MIMAT0005929	5223.4	4744.1	4983.8	338.9	533.3	382.0	457.6	107.0
hsa-miR-1276	MIMAT0005930	123.3	N.D.	-	-	18.9	20.6	19.7	1.2
hsa-miR-1280	MIMAT0005946	8420.1	9941.1	9180.6	1075.6	1887.3	1314.2	1600.8	405.3
hsa-miR-1281	MIMAT0005939	392.1	589.8	491.0	139.8	131.4	84.2	107.8	33.4
hsa-miR-1284	MIMAT0005941	N.D.	70.9	-	-	19.4	15.5	17.4	2.8
hsa-miR-1285	MIMAT0005876	124.6	242.2	183.4	83.1	12.6	17.7	15.2	3.6
hsa-miR-1286	MIMAT0005877	61.5	50.0	55.7	8.2	N.D.	N.D.	-	-
hsa-miR-1287	MIMAT0005878	156.7	104.9	130.8	36.6	16.0	18.5	17.3	1.7
hsa-miR-1288	MIMAT0005942	38.8	67.7	53.2	20.4	26.3	24.5	25.4	1.3
hsa-miR-1289	MIMAT0005879	N.D.	41.8	-	-	10.7	21.9	16.3	7.9
hsa-miR-1290	MIMAT0005880	103.4	281.1	192.2	125.6	18.9	23.9	21.4	3.6
hsa-miR-1291	MIMAT0005881	65.4	59.3	62.3	4.2	29.9	26.8	28.3	2.2
hsa-miR-1292	MIMAT0005943	223.7	146.2	184.9	54.8	20.0	16.6	18.3	2.4
hsa-miR-129-5p	MIMAT0000242	40.9	111.4	76.1	49.8	17.3	N.D.	-	-
hsa-miR-1296	MIMAT0005794	71.9	150.7	111.3	55.7	48.6	27.3	38.0	15.0
hsa-miR-1298	MIMAT0005800	41.5	114.1	77.8	51.3	11.5	19.7	15.6	5.8
hsa-miR-1301	MIMAT0005797	N.D.	N.D.	-	-	16.1	22.6	19.4	4.6
hsa-miR-1303	MIMAT0005891	N.D.	353.2	-	-	32.0	26.8	29.4	3.7
hsa-miR-1306	MIMAT0005950	52.7	102.6	77.7	35.2	27.6	15.5	21.6	8.6
hsa-miR-1307	MIMAT0005951	459.5	705.9	582.7	174.2	550.4	320.0	435.2	162.9
hsa-miR-130a	MIMAT0000425	N.D.	N.D.	-	-	134.6	20.3	77.4	80.8
hsa-miR-130b*	MIMAT0004680	55.3	68.4	61.8	9.2	19.0	N.D.	-	-
hsa-miR-1321	MIMAT0005952	N.D.	74.8	-	-	21.9	17.0	19.5	3.4
hsa-miR-134	MIMAT0000447	N.D.	82.2	-	-	23.8	25.9	24.9	1.5
hsa-miR-135a*	MIMAT0004595	283.3	1646.0	964.7	963.6	162.0	105.6	133.8	39.9
hsa-miR-138-1*	MIMAT0004607	69.1	89.8	79.5	14.7	22.8	25.5	24.2	1.9
hsa-miR-139-3p	MIMAT0004552	1613.6	443.1	1028.3	827.7	84.8	63.5	74.1	15.0
hsa-miR-140-3p	MIMAT0004597	N.D.	78.1	-	-	401.2	79.9	240.5	227.1
hsa-miR-141*	MIMAT0004598	N.D.	N.D.	-	-	19.7	26.7	23.2	4.9
hsa-miR-143*	MIMAT0004599	N.D.	N.D.	-	-	12.1	16.5	14.3	3.1
hsa-miR-145	MIMAT0000437	N.D.	N.D.	-	-	23.1	18.4	20.7	3.3
hsa-miR-1468	MIMAT0006789	67.8	61.2	64.5	4.7	23.3	18.3	20.8	3.6
hsa-miR-1469	MIMAT0007347	7326.9	2980.4	5153.6	3073.5	5236.9	2839.6	4038.2	1695.2
hsa-miR-146a	MIMAT0000449	78.1	2546.7	1312.4	1745.6	86.6	N.D.	-	-
hsa-miR-146b-3p	MIMAT0004766	N.D.	114.1	-	-	25.3	19.1	22.2	4.4
hsa-miR-146b-5p	MIMAT0002809	90.0	307.5	198.8	153.8	15.8	N.D.	-	-
hsa-miR-147	MIMAT0000251	222.3	106.8	164.5	81.7	35.1	21.7	28.4	9.5
hsa-miR-1470	MIMAT0007348	105.7	73.8	89.8	22.5	58.2	30.8	44.5	19.4
hsa-miR-1471	MIMAT0007349	168.4	249.8	209.1	57.6	48.5	39.0	43.7	6.7
hsa-miR-149	MIMAT0000450	133.0	194.9	163.9	43.8	70.3	41.2	55.8	20.6
hsa-miR-149*	MIMAT0004609	3581.5	6515.5	5048.5	2074.6	2152.6	1932.3	2042.5	155.7
hsa-miR-150	MIMAT0000451	N.D.	862.5	-	-	63.0	27.0	45.0	25.4
hsa-miR-150*	MIMAT0004610	128.5	239.4	183.9	78.4	19.8	15.6	17.7	2.9
hsa-miR-151-3p	MIMAT0000757	56.4	90.9	73.7	24.4	117.6	29.0	73.3	62.7
hsa-miR-151-5p	MIMAT0004697	N.D.	136.5	-	-	63.8	20.0	41.9	31.0

hsa-miR-1537	MIMAT0007399	49.6	40.0	44.8	6.8	N.D.	N.D.	-	-
hsa-miR-1538	MIMAT0007400	152.4	184.9	168.6	23.0	97.5	66.9	82.2	21.6
hsa-miR-1539	MIMAT0007401	190.4	211.4	200.9	14.9	100.0	54.0	77.0	32.6
hsa-miR-154	MIMAT0000452	N.D.	47.7	-	-	19.2	24.7	22.0	3.9
hsa-miR-15a	MIMAT0000068	N.D.	179.9	-	-	237.0	17.4	127.2	155.3
hsa-miR-15b	MIMAT0000417	N.D.	695.0	-	-	1055.5	98.4	577.0	676.8
hsa-miR-16	MIMAT0000069	N.D.	1131.7	-	-	1141.4	30.5	585.9	785.5
hsa-miR-16-2*	MIMAT0004518	N.D.	68.5	-	-	22.9	16.6	19.8	4.4
hsa-miR-17	MIMAT0000070	N.D.	174.1	-	-	501.3	43.9	272.6	323.4
hsa-miR-181a	MIMAT0000256	N.D.	183.5	-	-	66.5	31.2	48.8	24.9
hsa-miR-181a*	MIMAT0000270	54.3	N.D.	-	-	13.5	21.9	17.7	5.9
hsa-miR-181a-2*	MIMAT0004558	N.D.	56.3	-	-	22.3	28.5	25.4	4.4
hsa-miR-181d	MIMAT0002821	N.D.	N.D.	-	-	13.5	15.6	14.6	1.4
hsa-miR-182	MIMAT0000259	46.4	104.9	75.6	41.4	N.D.	18.4	-	-
hsa-miR-1825	MIMAT0006765	252.8	333.8	293.3	57.3	115.4	73.2	94.3	29.9
hsa-miR-184	MIMAT0000454	1173.4	2060.0	1616.7	626.9	21.6	17.1	19.3	3.2
hsa-miR-185	MIMAT0000455	N.D.	82.1	-	-	365.0	62.2	213.6	214.1
hsa-miR-185*	MIMAT0004611	191.3	151.2	171.3	28.4	31.5	30.0	30.7	1.1
hsa-miR-186	MIMAT0000456	N.D.	N.D.	-	-	80.3	16.1	48.2	45.4
hsa-miR-187	MIMAT0000262	50.9	111.1	81.0	42.5	174.1	44.4	109.2	91.8
hsa-miR-187*	MIMAT0004561	2408.4	2609.9	2509.1	142.5	1145.1	516.2	830.6	444.7
hsa-miR-188-5p	MIMAT0000457	56.8	97.7	77.3	28.9	21.9	19.1	20.5	2.0
hsa-miR-18a	MIMAT0000072	N.D.	N.D.	-	-	123.9	14.1	69.0	77.7
hsa-miR-18a*	MIMAT0002891	69.0	71.7	70.3	1.9	30.5	27.6	29.1	2.1
hsa-miR-18b*	MIMAT0004751	92.7	110.2	101.4	12.3	38.7	29.4	34.0	6.6
hsa-miR-1908	MIMAT0007881	15340.5	24685.4	20012.9	6607.8	1519.8	857.2	1188.5	468.5
hsa-miR-1909	MIMAT0007883	767.6	1284.1	1025.8	365.2	1029.4	804.8	917.1	158.8
hsa-miR-1909*	MIMAT0007882	192.6	213.3	202.9	14.6	84.4	51.6	68.0	23.2
hsa-miR-191	MIMAT0000440	N.D.	427.6	-	-	508.0	111.9	309.9	280.1
hsa-miR-1910	MIMAT0007884	238.6	299.6	269.1	43.1	146.4	75.9	111.1	49.8
hsa-miR-1911	MIMAT0007885	N.D.	71.5	-	-	15.4	15.2	15.3	0.1
hsa-miR-1911*	MIMAT0007886	42.5	79.0	60.8	25.8	18.7	21.5	20.1	2.0
hsa-miR-1913	MIMAT0007888	264.8	434.6	349.7	120.0	187.4	116.5	152.0	50.1
hsa-miR-1914	MIMAT0007889	60.3	133.7	97.0	51.9	27.2	26.9	27.0	0.2
hsa-miR-1914*	MIMAT0007890	786.7	755.2	770.9	22.2	219.1	110.7	164.9	76.6
hsa-miR-1915	MIMAT0007892	2587.9	2780.5	2684.2	136.1	2193.3	1024.6	1608.9	826.4
hsa-miR-192	MIMAT0000222	N.D.	92.8	-	-	48.9	23.7	36.3	17.8
hsa-miR-193a-5p	MIMAT0004614	129.8	112.0	120.9	12.6	39.6	24.3	31.9	10.9
hsa-miR-193b	MIMAT0002819	115.2	203.8	159.5	62.6	51.5	36.1	43.8	10.9
hsa-miR-193b*	MIMAT0004767	325.5	454.2	389.9	91.0	47.5	35.9	41.7	8.1
hsa-miR-194*	MIMAT0004671	82.8	90.3	86.6	5.3	76.2	58.5	67.3	12.5
hsa-miR-197	MIMAT0000227	200.5	389.1	294.8	133.4	200.3	74.1	137.2	89.3
hsa-miR-1972	MIMAT0009447	103.6	307.0	205.3	143.8	16.2	23.8	20.0	5.4
hsa-miR-1976	MIMAT0009451	352.9	586.7	469.8	165.3	166.3	76.9	121.6	63.2
hsa-miR-198	MIMAT0000228	66.3	149.9	108.1	59.2	16.1	N.D.	-	-
hsa-miR-199a-5p	MIMAT0000231	102.3	194.9	148.6	65.5	41.8	22.2	32.0	13.8
hsa-miR-199b-5p	MIMAT0000263	146.7	114.1	130.4	23.1	21.5	N.D.	-	-
hsa-miR-19a	MIMAT0000073	N.D.	N.D.	-	-	186.3	32.7	109.5	108.6
hsa-miR-19b	MIMAT0000074	N.D.	179.0	-	-	695.6	172.5	434.0	369.8
hsa-miR-200a	MIMAT0000682	63.5	1640.6	852.1	1115.2	9.7	N.D.	-	-
hsa-miR-200b	MIMAT0000318	39.8	1281.8	660.8	878.3	N.D.	N.D.	-	-
hsa-miR-200b*	MIMAT0004571	39.0	63.7	51.4	17.5	N.D.	N.D.	-	-
hsa-miR-200c	MIMAT0000617	119.3	1327.0	723.1	854.0	N.D.	N.D.	-	-
hsa-miR-202	MIMAT0002811	125.1	80.7	102.9	31.4	N.D.	N.D.	-	-
hsa-miR-203	MIMAT0000264	184.4	990.1	587.3	569.7	22.1	18.7	20.4	2.4
hsa-miR-204	MIMAT0000265	98.2	371.2	234.7	193.0	23.5	19.9	21.7	2.5

hsa-miR-205	MIMAT0000266	96.5	770.5	433.5	476.6	31.2	16.1	23.6	10.6
hsa-miR-20a	MIMAT0000075	N.D.	144.0	-	-	358.7	23.9	191.3	236.7
hsa-miR-20b	MIMAT0001413	N.D.	86.2	-	-	330.6	24.7	177.7	216.3
hsa-miR-210	MIMAT0000267	43.3	83.7	63.5	28.5	268.6	65.0	166.8	144.0
hsa-miR-211	MIMAT0000268	133.8	117.9	125.8	11.3	36.3	35.1	35.7	0.8
hsa-miR-2110	MIMAT0010133	257.6	153.9	205.7	73.3	42.4	37.7	40.1	3.3
hsa-miR-2113	MIMAT0009206	113.3	273.0	193.2	112.9	23.2	N.D.	-	-
hsa-miR-2114	MIMAT0011156	N.D.	70.2	-	-	13.3	14.6	13.9	0.9
hsa-miR-2116*	MIMAT0011161	73.1	154.6	113.8	57.6	36.0	23.3	29.6	9.0
hsa-miR-212	MIMAT0000269	113.3	75.8	94.5	26.5	25.8	14.1	19.9	8.3
hsa-miR-214	MIMAT0000271	72.4	134.2	103.3	43.7	45.7	24.6	35.2	14.9
hsa-miR-214*	MIMAT0004564	44.7	58.2	51.5	9.5	25.3	N.D.	-	-
hsa-miR-218-2*	MIMAT0004566	N.D.	46.4	-	-	11.9	19.4	15.7	5.3
hsa-miR-22	MIMAT0000077	N.D.	438.8	-	-	911.2	227.5	569.4	483.4
hsa-miR-221	MIMAT0000278	40.1	387.1	213.6	245.3	845.6	65.3	455.4	551.7
hsa-miR-222	MIMAT0000279	N.D.	180.2	-	-	131.8	15.6	73.7	82.1
hsa-miR-223	MIMAT0000280	102.4	13183.7	6643.1	9249.9	10899.1	296.7	5597.9	7497.0
hsa-miR-223*	MIMAT0004570	42.2	64.4	53.3	15.7	12.8	19.1	16.0	4.4
hsa-miR-224*	MIMAT0009198	183.6	51.0	117.3	93.7	10.3	N.D.	-	-
hsa-miR-2276	MIMAT0011775	282.8	247.9	265.3	24.6	24.5	21.4	22.9	2.2
hsa-miR-2277-3p	MIMAT0011777	207.7	152.1	179.9	39.3	57.5	30.1	43.8	19.4
hsa-miR-2278	MIMAT0011778	410.0	329.2	369.6	57.1	74.3	37.1	55.7	26.4
hsa-miR-2355-5p	MIMAT0016895	61.3	84.4	72.9	16.3	46.2	35.3	40.7	7.7
hsa-miR-23a	MIMAT0000078	173.7	2386.8	1280.2	1564.9	2251.3	124.8	1188.1	1503.7
hsa-miR-23a*	MIMAT0004496	89.1	373.9	231.5	201.4	17.5	N.D.	-	-
hsa-miR-23b	MIMAT0000418	150.6	2081.3	1115.9	1365.2	2173.8	93.9	1133.9	1470.7
hsa-miR-24	MIMAT0000080	467.2	2113.3	1290.2	1164.0	819.7	136.1	477.9	483.4
hsa-miR-25	MIMAT0000081	N.D.	254.5	-	-	240.7	35.2	137.9	145.3
hsa-miR-25*	MIMAT0004498	276.0	143.4	209.7	93.8	13.1	N.D.	-	-
hsa-miR-26a	MIMAT0000082	90.8	1717.4	904.1	1150.2	191.2	N.D.	-	-
hsa-miR-26b*	MIMAT0004500	69.3	154.4	111.8	60.2	40.1	32.1	36.1	5.7
hsa-miR-27a	MIMAT0000084	N.D.	416.7	-	-	530.7	19.6	275.1	361.4
hsa-miR-27b	MIMAT0000419	N.D.	215.4	-	-	108.3	25.0	66.7	58.9
hsa-miR-2861	MIMAT0013802	14951.8	30825.0	22888.4	11224.0	20513.9	16293.6	18403.7	2984.2
hsa-miR-296-3p	MIMAT0004679	362.6	213.6	288.1	105.3	46.8	41.9	44.4	3.5
hsa-miR-296-5p	MIMAT0000690	504.6	835.8	670.2	234.2	344.9	239.3	292.1	74.7
hsa-miR-298	MIMAT0004901	70.1	68.6	69.3	1.1	N.D.	19.7	-	-
hsa-miR-299-5p	MIMAT0002890	60.5	106.2	83.4	32.4	22.7	21.1	21.9	1.1
hsa-miR-29a	MIMAT0000086	N.D.	664.9	-	-	92.0	32.8	62.4	41.8
hsa-miR-29c	MIMAT0000681	N.D.	259.3	-	-	108.9	26.2	67.5	58.5
hsa-miR-29c*	MIMAT0004673	N.D.	58.5	-	-	23.3	19.3	21.3	2.8
hsa-miR-301a	MIMAT0000688	N.D.	N.D.	-	-	47.7	15.8	31.7	22.5
hsa-miR-302c	MIMAT0000717	N.D.	54.9	-	-	11.7	23.4	17.6	8.3
hsa-miR-302c*	MIMAT0000716	277.9	81.1	179.5	139.2	20.8	17.1	18.9	2.6
hsa-miR-302d	MIMAT0000718	N.D.	N.D.	-	-	17.1	14.3	15.7	1.9
hsa-miR-302f	MIMAT0005932	N.D.	N.D.	-	-	13.8	21.1	17.4	5.2
hsa-miR-3065-3p	MIMAT0015378	63.0	80.4	71.7	12.3	13.0	20.4	16.7	5.2
hsa-miR-3074	MIMAT0015027	N.D.	65.4	-	-	35.0	22.5	28.7	8.8
hsa-miR-30b	MIMAT0000420	50.6	267.9	159.3	153.6	172.3	22.0	97.2	106.2
hsa-miR-30b*	MIMAT0004589	38.6	62.2	50.4	16.7	15.3	N.D.	-	-
hsa-miR-30c	MIMAT0000244	54.9	381.2	218.1	230.8	83.0	22.4	52.7	42.9
hsa-miR-30c-1*	MIMAT0004674	N.D.	201.5	-	-	32.4	19.0	25.7	9.5
hsa-miR-30c-2*	MIMAT0004550	1430.5	67.9	749.2	963.5	13.3	N.D.	-	-
hsa-miR-30d	MIMAT0000245	46.7	285.7	166.2	168.9	691.6	88.8	390.2	426.2
hsa-miR-30e	MIMAT0000692	N.D.	107.0	-	-	186.9	26.7	106.8	113.3
hsa-miR-30e*	MIMAT0000693	N.D.	85.2	-	-	29.5	19.2	24.3	7.2

hsa-miR-3119	MIMAT0014981	58.0	61.2	59.6	2.2	12.6	N.D.	-	-
hsa-miR-3120	MIMAT0014982	N.D.	N.D.	-	-	12.1	16.8	14.4	3.3
hsa-miR-3122	MIMAT0014984	334.6	372.4	353.5	26.8	22.7	15.3	19.0	5.3
hsa-miR-3124	MIMAT0014986	633.7	182.6	408.2	319.0	27.2	26.6	26.9	0.4
hsa-miR-3125	MIMAT0014988	62.6	81.8	72.2	13.6	13.2	N.D.	-	-
hsa-miR-3126-3p	MIMAT0015377	55.1	87.0	71.0	22.5	23.0	19.8	21.4	2.3
hsa-miR-3126-5p	MIMAT0014989	135.8	439.5	287.7	214.8	15.7	19.6	17.6	2.7
hsa-miR-3127	MIMAT0014990	59.6	93.5	76.5	24.0	N.D.	N.D.	-	-
hsa-miR-3130-3p	MIMAT0014994	137.5	83.3	110.4	38.3	17.5	N.D.	-	-
hsa-miR-3130-5p	MIMAT0014995	119.8	192.0	155.9	51.0	58.6	22.2	40.4	25.7
hsa-miR-3131	MIMAT0014996	913.5	564.1	738.8	247.1	93.8	112.4	103.1	13.1
hsa-miR-3132	MIMAT0014997	97.7	98.5	98.1	0.6	16.7	N.D.	-	-
hsa-miR-3137	MIMAT0015005	91.1	101.7	96.4	7.6	11.1	23.2	17.1	8.5
hsa-miR-3138	MIMAT0015006	85.7	71.7	78.7	9.9	17.8	N.D.	-	-
hsa-miR-3141	MIMAT0015010	1420.3	1768.0	1594.2	245.9	956.6	506.9	731.8	317.9
hsa-miR-3144-5p	MIMAT0015014	69.0	65.7	67.4	2.3	12.3	21.6	17.0	6.5
hsa-miR-3147	MIMAT0015019	189.1	240.9	215.0	36.6	34.8	15.4	25.1	13.7
hsa-miR-3149	MIMAT0015022	39.2	N.D.	-	-	14.4	14.1	14.3	0.2
hsa-miR-3150	MIMAT0015023	70.4	225.5	148.0	109.7	22.0	N.D.	-	-
hsa-miR-3150b	MIMAT0018194	142.0	118.7	130.4	16.5	22.4	14.6	18.5	5.6
hsa-miR-3151	MIMAT0015024	414.5	518.0	466.2	73.2	74.7	53.2	64.0	15.2
hsa-miR-3153	MIMAT0015026	367.0	107.7	237.4	183.4	20.8	14.1	17.5	4.7
hsa-miR-3154	MIMAT0015028	459.4	528.7	494.1	49.0	107.2	70.2	88.7	26.2
hsa-miR-3155	MIMAT0015029	56.1	63.1	59.6	4.9	10.6	N.D.	-	-
hsa-miR-3156	MIMAT0015030	128.0	97.6	112.8	21.5	14.0	22.7	18.3	6.1
hsa-miR-3158	MIMAT0015032	77.6	110.8	94.2	23.5	33.0	17.5	25.2	10.9
hsa-miR-3162	MIMAT0015036	317.1	915.7	616.4	423.2	73.9	46.8	60.3	19.2
hsa-miR-3163	MIMAT0015037	42.9	61.6	52.3	13.2	N.D.	N.D.	-	-
hsa-miR-3173	MIMAT0015048	54.9	78.3	66.6	16.5	20.7	25.5	23.1	3.4
hsa-miR-3174	MIMAT0015051	N.D.	40.2	-	-	11.0	14.0	12.5	2.1
hsa-miR-3175	MIMAT0015052	525.3	381.2	453.2	101.9	42.0	19.8	30.9	15.7
hsa-miR-3176	MIMAT0015053	71.8	129.4	100.6	40.7	30.8	N.D.	-	-
hsa-miR-3177	MIMAT0015054	158.3	149.0	153.7	6.6	24.4	33.6	29.0	6.5
hsa-miR-3178	MIMAT0015055	10211.0	6741.4	8476.2	2453.3	4955.8	3049.8	4002.8	1347.7
hsa-miR-3180	MIMAT0018178	1332.3	1461.8	1397.0	91.5	1791.8	1001.2	1396.5	559.0
hsa-miR-3180-3p	MIMAT0015058	1278.3	919.0	1098.7	254.1	866.4	490.7	678.6	265.7
hsa-miR-3180-5p	MIMAT0015057	74.1	132.8	103.5	41.5	76.6	53.6	65.1	16.2
hsa-miR-3181	MIMAT0015061	40.0	50.8	45.4	7.7	15.7	19.3	17.5	2.5
hsa-miR-3184	MIMAT0015064	210.2	185.2	197.7	17.7	44.6	33.6	39.1	7.8
hsa-miR-3185	MIMAT0015065	658.9	739.0	699.0	56.7	97.3	43.8	70.6	37.8
hsa-miR-3186-5p	MIMAT0015067	68.0	90.0	79.0	15.6	19.0	18.4	18.7	0.4
hsa-miR-3187	MIMAT0015069	68.5	114.8	91.6	32.7	14.3	16.8	15.5	1.8
hsa-miR-3188	MIMAT0015070	2329.2	607.8	1468.5	1217.3	184.9	119.2	152.1	46.5
hsa-miR-3189	MIMAT0015071	76.8	46.2	61.5	21.6	14.4	N.D.	-	-
hsa-miR-3190	MIMAT0015073	221.6	152.6	187.1	48.8	38.4	20.6	29.5	12.6
hsa-miR-3191	MIMAT0015075	205.4	256.1	230.7	35.9	38.1	37.8	38.0	0.3
hsa-miR-3192	MIMAT0015076	353.6	178.4	266.0	123.9	34.0	N.D.	-	-
hsa-miR-3194	MIMAT0015078	170.0	357.1	263.5	132.3	42.1	21.9	32.0	14.3
hsa-miR-3195	MIMAT0015079	2669.5	2453.8	2561.6	152.6	419.3	343.8	381.6	53.4
hsa-miR-3196	MIMAT0015080	3715.3	6537.1	5126.2	1995.3	7693.9	5265.9	6479.9	1716.8
hsa-miR-3197	MIMAT0015082	30306.9	3263.7	16785.3	19122.4	999.8	698.7	849.2	212.8
hsa-miR-3198	MIMAT0015083	77.3	107.6	92.5	21.4	15.7	27.5	21.6	8.4
hsa-miR-3200-3p	MIMAT0015085	52.7	66.1	59.4	9.5	16.2	18.0	17.1	1.3
hsa-miR-3202	MIMAT0015089	165.7	57.3	111.5	76.7	N.D.	N.D.	-	-
hsa-miR-320a	MIMAT0000510	201.6	170.1	185.8	22.3	65.9	34.5	50.2	22.2
hsa-miR-320b	MIMAT0005792	117.3	123.6	120.4	4.5	70.0	44.5	57.3	18.0

hsa-miR-320c	MIMAT0005793	N.D.	100.2	-	-	57.0	33.3	45.2	16.7
hsa-miR-320d	MIMAT0006764	67.6	96.1	81.8	20.2	55.4	32.6	44.0	16.1
hsa-miR-320e	MIMAT0015072	N.D.	66.9	-	-	38.4	31.8	35.1	4.7
hsa-miR-323-5p	MIMAT0004696	85.2	110.9	98.0	18.2	20.5	N.D.	-	-
hsa-miR-323b-3p	MIMAT0015050	N.D.	N.D.	-	-	12.6	17.3	14.9	3.3
hsa-miR-323b-5p	MIMAT0001630	N.D.	73.7	-	-	17.3	22.3	19.8	3.5
hsa-miR-324-3p	MIMAT0000762	N.D.	149.2	-	-	41.0	24.1	32.5	11.9
hsa-miR-326	MIMAT0000756	160.7	145.6	153.2	10.7	105.1	42.2	73.7	44.5
hsa-miR-328	MIMAT0000752	263.7	317.1	290.4	37.8	138.6	85.8	112.2	37.3
hsa-miR-330-3p	MIMAT0000751	114.6	69.1	91.9	32.1	20.7	24.6	22.6	2.8
hsa-miR-330-5p	MIMAT0004693	N.D.	124.6	-	-	65.2	26.1	45.7	27.6
hsa-miR-331-3p	MIMAT0000760	N.D.	83.5	-	-	64.7	15.8	40.2	34.6
hsa-miR-337-3p	MIMAT0000754	54.4	67.2	60.8	9.1	18.2	N.D.	-	-
hsa-miR-339-5p	MIMAT0000764	39.0	152.4	95.7	80.2	36.6	19.9	28.3	11.8
hsa-miR-340*	MIMAT0000750	102.8	68.5	85.7	24.2	15.5	N.D.	-	-
hsa-miR-342-3p	MIMAT0000753	98.3	178.5	138.4	56.7	55.4	26.3	40.8	20.6
hsa-miR-342-5p	MIMAT0004694	299.3	141.7	220.5	111.4	N.D.	N.D.	-	-
hsa-miR-345	MIMAT0000772	59.2	46.7	53.0	8.9	9.7	N.D.	-	-
hsa-miR-346	MIMAT0000773	131.6	260.7	196.2	91.3	89.5	40.8	65.1	34.5
hsa-miR-34a	MIMAT0000255	90.2	255.8	173.0	117.1	28.5	23.6	26.0	3.5
hsa-miR-34b	MIMAT0004676	89.9	58.3	74.1	22.4	18.8	20.5	19.7	1.2
hsa-miR-34c-3p	MIMAT0004677	75.9	83.3	79.6	5.2	35.4	27.2	31.3	5.8
hsa-miR-3605-3p	MIMAT0017982	141.6	258.8	200.2	82.9	58.5	34.5	46.5	17.0
hsa-miR-3605-5p	MIMAT0017981	101.3	116.1	108.7	10.5	N.D.	N.D.	-	-
hsa-miR-3610	MIMAT0017987	562.2	380.4	471.3	128.5	16.1	15.3	15.7	0.5
hsa-miR-3612	MIMAT0017989	46.0	42.9	44.5	2.2	N.D.	N.D.	-	-
hsa-miR-361-3p	MIMAT0004682	61.2	120.5	90.9	41.9	39.3	21.6	30.4	12.6
hsa-miR-3614-5p	MIMAT0017992	126.9	176.7	151.8	35.2	69.1	47.8	58.4	15.1
hsa-miR-3615	MIMAT0017994	59.8	108.1	83.9	34.1	16.8	N.D.	-	-
hsa-miR-361-5p	MIMAT0000703	N.D.	135.5	-	-	145.7	29.7	87.7	82.0
hsa-miR-3616-3p	MIMAT0017996	775.1	1471.6	1123.3	492.5	40.1	23.0	31.5	12.1
hsa-miR-3619	MIMAT0017999	92.2	110.9	101.5	13.2	18.7	N.D.	-	-
hsa-miR-3620	MIMAT0018001	153.4	219.9	186.6	47.0	67.7	33.5	50.6	24.2
hsa-miR-3621	MIMAT0018002	6751.6	22848.0	14799.8	11381.9	2972.3	1926.4	2449.3	739.6
hsa-miR-3622a-3p	MIMAT0018004	159.0	240.3	199.6	57.4	80.1	47.9	64.0	22.8
hsa-miR-3622a-5p	MIMAT0018003	358.3	413.9	386.1	39.3	68.1	37.6	52.9	21.6
hsa-miR-3622b-3p	MIMAT0018006	77.6	160.2	118.9	58.4	36.1	25.5	30.8	7.5
hsa-miR-3622b-5p	MIMAT0018005	319.5	416.4	367.9	68.5	37.6	19.7	28.6	12.6
hsa-miR-363*	MIMAT0003385	42.4	60.4	51.4	12.8	13.2	18.4	15.8	3.7
hsa-miR-3646	MIMAT0018065	144.8	123.1	134.0	15.3	46.3	26.1	36.2	14.2
hsa-miR-3648	MIMAT0018068	14545.5	16620.1	15582.8	1466.9	2892.5	1154.9	2023.7	1228.6
hsa-miR-3649	MIMAT0018069	67.7	80.8	74.3	9.2	15.4	N.D.	-	-
hsa-miR-365	MIMAT0000710	52.4	56.6	54.5	3.0	18.2	N.D.	-	-
hsa-miR-365*	MIMAT0009199	129.3	134.2	131.8	3.4	18.0	15.8	16.9	1.5
hsa-miR-3650	MIMAT0018070	66.4	63.9	65.1	1.8	17.1	N.D.	-	-
hsa-miR-3651	MIMAT0018071	59.8	77.8	68.8	12.7	20.2	N.D.	-	-
hsa-miR-3652	MIMAT0018072	315.7	272.7	294.2	30.4	37.8	21.0	29.4	11.8
hsa-miR-3654	MIMAT0018074	56.4	76.6	66.5	14.3	18.0	N.D.	-	-
hsa-miR-3655	MIMAT0018075	45.2	82.5	63.9	26.4	17.1	N.D.	-	-
hsa-miR-3656	MIMAT0018076	11275.1	21992.2	16633.6	7578.2	6991.6	3967.0	5479.3	2138.7
hsa-miR-3659	MIMAT0018080	50.7	87.1	68.9	25.7	26.5	21.6	24.1	3.5
hsa-miR-3660	MIMAT0018081	49.1	57.1	53.1	5.6	10.8	N.D.	-	-
hsa-miR-3661	MIMAT0018082	102.4	82.4	92.4	14.2	12.0	N.D.	-	-
hsa-miR-3663-3p	MIMAT0018085	868.6	706.1	787.3	114.9	1798.3	1148.6	1473.5	459.4
hsa-miR-3663-5p	MIMAT0018084	195.2	245.1	220.1	35.3	72.6	52.7	62.6	14.1
hsa-miR-3665	MIMAT0018087	15602.1	21909.3	18755.7	4459.9	17820.6	7953.9	12887.2	6976.8

hsa-miR-3666	MIMAT0018088	N.D.	N.D.	-	-	10.2	22.2	16.2	8.5
hsa-miR-3667-3p	MIMAT0018090	108.8	155.7	132.3	33.2	35.8	23.4	29.6	8.8
hsa-miR-3667-5p	MIMAT0018089	52.0	66.2	59.1	10.0	15.8	14.1	15.0	1.3
hsa-miR-3675-3p	MIMAT0018099	76.4	205.6	141.0	91.3	63.0	42.3	52.7	14.7
hsa-miR-3676	MIMAT0018100	120.7	160.1	140.4	27.9	41.9	27.8	34.9	10.0
hsa-miR-3678-3p	MIMAT0018103	475.1	420.2	447.7	38.8	26.8	N.D.	-	-
hsa-miR-3678-5p	MIMAT0018102	49.9	43.4	46.7	4.6	13.4	N.D.	-	-
hsa-miR-3679-3p	MIMAT0018105	266.5	397.5	332.0	92.7	156.0	89.7	122.9	46.8
hsa-miR-3679-5p	MIMAT0018104	952.5	2188.5	1570.5	874.0	110.9	57.5	84.2	37.8
hsa-miR-3682	MIMAT0018110	125.0	177.9	151.5	37.4	27.1	N.D.	-	-
hsa-miR-3685	MIMAT0018113	66.4	59.9	63.2	4.6	20.4	27.7	24.0	5.2
hsa-miR-3687	MIMAT0018115	42.6	128.7	85.6	60.9	10.2	N.D.	-	-
hsa-miR-3689a-3p	MIMAT0018118	65.7	81.1	73.4	10.9	21.1	16.9	19.0	3.0
hsa-miR-3689a-5p,<br>hsa-miR-3689b	MIMAT0018117,<br>MIMAT0018180	N.D.	N.D.	-	-	10.4	20.5	15.4	7.1
hsa-miR-3689b*	MIMAT0018181	84.5	90.8	87.6	4.5	26.7	27.1	26.9	0.3
hsa-miR-3690	MIMAT0018119	72.6	91.7	82.2	13.5	15.5	16.7	16.1	0.9
hsa-miR-3691	MIMAT0018120	69.5	59.8	64.6	6.9	15.8	29.6	22.7	9.8
hsa-miR-3692*	MIMAT0018121	114.4	89.3	101.9	17.7	10.1	20.3	15.2	7.2
hsa-miR-370	MIMAT0000722	1005.4	550.1	777.7	321.9	60.5	44.3	52.4	11.4
hsa-miR-3713	MIMAT0018164	38.9	N.D.	-	-	10.4	19.7	15.1	6.6
hsa-miR-3714	MIMAT0018165	159.7	143.3	151.5	11.6	34.5	28.9	31.7	3.9
hsa-miR-371-5p	MIMAT0004687	321.9	182.3	252.1	98.7	81.4	63.0	72.2	13.0
hsa-miR-372	MIMAT0000724	N.D.	50.7	-	-	20.4	16.0	18.2	3.1
hsa-miR-373	MIMAT0000726	52.7	N.D.	-	-	11.0	14.1	12.5	2.2
hsa-miR-373*	MIMAT0000725	61.7	57.9	59.8	2.7	31.9	33.9	32.9	1.4
hsa-miR-375	MIMAT0000728	58.4	119.7	89.0	43.3	12.0	N.D.	-	-
hsa-miR-376a	MIMAT0000729	N.D.	N.D.	-	-	22.0	18.1	20.1	2.7
hsa-miR-376c	MIMAT0000720	N.D.	N.D.	-	-	24.6	15.7	20.2	6.3
hsa-miR-377	MIMAT0000730	38.7	N.D.	-	-	25.4	19.4	22.4	4.3
hsa-miR-378	MIMAT0000732	N.D.	101.8	-	-	48.8	21.7	35.2	19.1
hsa-miR-378*	MIMAT0000731	49.2	62.3	55.8	9.3	20.3	N.D.	-	-
hsa-miR-378b	MIMAT0014999	43.5	45.4	44.4	1.3	14.6	N.D.	-	-
hsa-miR-379	MIMAT0000733	43.7	60.8	52.3	12.1	20.1	17.6	18.8	1.7
hsa-miR-381	MIMAT0000736	103.1	63.3	83.2	28.2	41.5	27.4	34.5	10.0
hsa-miR-382	MIMAT0000737	N.D.	48.9	-	-	42.3	19.8	31.0	15.9
hsa-miR-383	MIMAT0000738	N.D.	N.D.	-	-	11.4	15.7	13.6	3.0
hsa-miR-3907	MIMAT0018179	58.4	66.9	62.6	6.0	22.4	24.0	23.2	1.1
hsa-miR-3908	MIMAT0018182	N.D.	N.D.	-	-	13.1	16.4	14.7	2.4
hsa-miR-3911	MIMAT0018185	119.6	100.3	110.0	13.7	17.0	N.D.	-	-
hsa-miR-3917	MIMAT0018191	462.1	438.2	450.1	16.9	90.7	71.7	81.2	13.4
hsa-miR-3918	MIMAT0018192	455.7	1337.6	896.7	623.6	52.5	22.4	37.5	21.3
hsa-miR-3925	MIMAT0018200	67.8	116.9	92.3	34.8	12.4	N.D.	-	-
hsa-miR-3926	MIMAT0018201	48.6	73.7	61.2	17.7	15.4	N.D.	-	-
hsa-miR-3928	MIMAT0018205	790.3	580.6	685.5	148.3	158.8	138.7	148.8	14.2
hsa-miR-3929	MIMAT0018206	48.7	60.0	54.4	8.0	20.8	N.D.	-	-
hsa-miR-3934	MIMAT0018349	153.8	313.5	233.7	112.9	50.6	40.0	45.3	7.5
hsa-miR-3935	MIMAT0018350	88.3	111.6	99.9	16.5	44.2	34.0	39.1	7.2
hsa-miR-3936	MIMAT0018351	404.6	174.0	289.3	163.0	13.1	19.2	16.1	4.3
hsa-miR-3937	MIMAT0018352	905.1	1624.4	1264.7	508.6	357.5	188.9	273.2	119.2
hsa-miR-3940	MIMAT0018356	282.2	315.2	298.7	23.4	87.8	67.1	77.5	14.7
hsa-miR-3941	MIMAT0018357	N.D.	N.D.	-	-	9.5	19.5	14.5	7.0
hsa-miR-3943	MIMAT0018359	N.D.	82.4	-	-	25.5	27.1	26.3	1.1
hsa-miR-3944	MIMAT0018360	163.6	189.8	176.7	18.6	59.6	44.9	52.3	10.4
hsa-miR-3945	MIMAT0018361	66.7	79.8	73.3	9.3	15.4	22.1	18.8	4.7
hsa-miR-409-3p	MIMAT0001639	42.2	470.2	256.2	302.6	54.6	19.8	37.2	24.6

hsa-miR-410	MIMAT0002171	50.5	N.D.	-	-	14.1	23.5	18.8	6.7
hsa-miR-412	MIMAT0002170	52.2	90.8	71.5	27.3	24.3	N.D.	-	-
hsa-miR-422a	MIMAT0001339	71.5	50.1	60.8	15.1	32.5	22.3	27.4	7.2
hsa-miR-423-5p	MIMAT0004748	622.3	307.6	464.9	222.5	417.7	178.0	297.8	169.5
hsa-miR-425	MIMAT0003393	N.D.	144.7	-	-	256.5	44.5	150.5	149.9
hsa-miR-4252	MIMAT0016886	98.3	80.4	89.3	12.7	27.5	30.9	29.2	2.4
hsa-miR-4253	MIMAT0016882	206.9	150.5	178.7	39.9	50.2	32.5	41.4	12.5
hsa-miR-4254	MIMAT0016884	85.2	126.4	105.8	29.1	22.4	18.1	20.2	3.1
hsa-miR-4257	MIMAT0016878	770.0	2052.9	1411.5	907.1	80.2	74.9	77.5	3.7
hsa-miR-4258	MIMAT0016879	278.5	231.1	254.8	33.5	157.1	100.2	128.6	40.2
hsa-miR-4259	MIMAT0016880	198.8	259.5	229.2	42.9	57.9	38.6	48.3	13.7
hsa-miR-4260	MIMAT0016881	331.4	517.9	424.7	131.9	31.5	23.8	27.6	5.5
hsa-miR-4261	MIMAT0016890	255.8	128.3	192.0	90.1	14.2	N.D.	-	-
hsa-miR-4262	MIMAT0016894	N.D.	N.D.	-	-	14.3	21.5	17.9	5.1
hsa-miR-4263	MIMAT0016898	40.0	46.2	43.1	4.4	N.D.	N.D.	-	-
hsa-miR-4265	MIMAT0016891	135.9	129.3	132.6	4.7	35.3	44.1	39.7	6.2
hsa-miR-4266	MIMAT0016892	N.D.	N.D.	-	-	17.2	15.3	16.2	1.3
hsa-miR-4267	MIMAT0016893	52.0	59.9	56.0	5.6	17.6	N.D.	-	-
hsa-miR-4268	MIMAT0016896	187.0	157.4	172.2	20.9	64.5	46.1	55.3	13.0
hsa-miR-4269	MIMAT0016897	94.8	127.1	111.0	22.8	39.1	29.0	34.1	7.1
hsa-miR-4270	MIMAT0016900	1469.2	1330.1	1399.7	98.4	430.8	341.1	385.9	63.4
hsa-miR-4271	MIMAT0016901	332.9	521.3	427.1	133.3	290.6	167.0	228.8	87.4
hsa-miR-4274	MIMAT0016906	156.4	202.0	179.2	32.3	51.8	48.6	50.2	2.3
hsa-miR-4276	MIMAT0016904	132.6	105.6	119.1	19.1	82.0	58.1	70.0	16.9
hsa-miR-4279	MIMAT0016909	331.0	446.8	388.9	81.9	125.8	77.1	101.5	34.4
hsa-miR-4280	MIMAT0016911	N.D.	N.D.	-	-	11.8	14.9	13.4	2.2
hsa-miR-4281	MIMAT0016907	11596.1	14419.0	13007.5	1996.1	5248.9	2945.4	4097.1	1628.8
hsa-miR-4283	MIMAT0016914	57.2	71.1	64.2	9.8	20.8	N.D.	-	-
hsa-miR-4285	MIMAT0016913	46.4	54.3	50.4	5.6	N.D.	N.D.	-	-
hsa-miR-4286	MIMAT0016916	99.7	1042.8	571.3	666.9	207.6	45.7	126.7	114.5
hsa-miR-4287	MIMAT0016917	42.4	84.4	63.4	29.7	10.8	N.D.	-	-
hsa-miR-4290	MIMAT0016921	283.9	369.9	326.9	60.8	117.3	70.8	94.1	32.9
hsa-miR-4291	MIMAT0016922	N.D.	54.0	-	-	24.4	17.1	20.7	5.2
hsa-miR-4292	MIMAT0016919	N.D.	N.D.	-	-	10.9	20.8	15.9	7.0
hsa-miR-4293	MIMAT0016848	47.1	50.4	48.8	2.4	10.0	N.D.	-	-
hsa-miR-4294	MIMAT0016849	4206.8	40342.8	22274.8	25552.0	6473.6	4528.9	5501.2	1375.2
hsa-miR-4295	MIMAT0016844	58.1	57.6	57.8	0.4	14.6	N.D.	-	-
hsa-miR-4296	MIMAT0016845	78.4	112.9	95.6	24.4	20.2	N.D.	-	-
hsa-miR-4297	MIMAT0016846	155.7	250.8	203.2	67.3	74.0	38.0	56.0	25.5
hsa-miR-4298	MIMAT0016852	629.5	605.6	617.6	16.9	128.1	70.8	99.5	40.5
hsa-miR-4299	MIMAT0016851	302.2	221.6	261.9	57.0	14.7	16.6	15.6	1.3
hsa-miR-4300	MIMAT0016853	147.6	61.1	104.3	61.1	15.9	21.1	18.5	3.7
hsa-miR-4304	MIMAT0016854	139.0	62.6	100.8	54.0	16.9	N.D.	-	-
hsa-miR-4305	MIMAT0016857	84.3	138.2	111.2	38.1	56.2	35.3	45.8	14.8
hsa-miR-4306	MIMAT0016858	70.7	63.7	67.2	5.0	205.0	45.1	125.1	113.1
hsa-miR-4308	MIMAT0016861	59.7	71.1	65.4	8.0	N.D.	14.9	-	-
hsa-miR-4310	MIMAT0016862	N.D.	50.9	-	-	19.4	17.3	18.3	1.4
hsa-miR-4311	MIMAT0016863	51.8	92.0	71.9	28.5	13.8	N.D.	-	-
hsa-miR-4312	MIMAT0016864	95.1	165.6	130.4	49.9	46.5	33.0	39.7	9.5
hsa-miR-4314	MIMAT0016868	64.2	76.6	70.4	8.8	12.1	17.3	14.7	3.7
hsa-miR-4315	MIMAT0016866	42.2	74.7	58.4	23.0	15.3	N.D.	-	-
hsa-miR-4316	MIMAT0016867	149.1	73.4	111.3	53.6	10.2	N.D.	-	-
hsa-miR-4318	MIMAT0016869	40.2	76.2	58.2	25.4	N.D.	N.D.	-	-
hsa-miR-4319	MIMAT0016870	69.1	75.7	72.4	4.7	18.2	21.1	19.6	2.0
hsa-miR-432	MIMAT0002814	N.D.	N.D.	-	-	18.0	16.3	17.1	1.2
hsa-miR-432*	MIMAT0002815	N.D.	119.6	-	-	40.0	15.4	27.7	17.4

hsa-miR-4321	MIMAT0016874	109.2	153.1	131.2	31.0	17.6	N.D.	-	-
hsa-miR-4322	MIMAT0016873	528.1	465.9	497.0	44.0	161.5	99.6	130.6	43.8
hsa-miR-4323	MIMAT0016875	176.1	299.1	237.6	87.0	96.4	48.4	72.4	33.9
hsa-miR-4324	MIMAT0016876	50.2	136.5	93.4	61.1	34.3	25.4	29.8	6.3
hsa-miR-4326	MIMAT0016888	151.3	185.7	168.5	24.3	53.1	36.0	44.6	12.1
hsa-miR-4327	MIMAT0016889	831.3	1538.3	1184.8	499.9	466.5	360.1	413.3	75.2
hsa-miR-4329	MIMAT0016923	102.4	101.6	102.0	0.6	30.9	17.0	23.9	9.9
hsa-miR-4330	MIMAT0016924	159.7	76.0	117.9	59.2	15.8	23.4	19.6	5.4
hsa-miR-449b*	MIMAT0009203	201.4	250.0	225.7	34.4	102.0	55.4	78.7	33.0
hsa-miR-449c*	MIMAT0013771	80.2	114.5	97.4	24.3	32.8	14.7	23.7	12.9
hsa-miR-451	MIMAT0001631	N.D.	N.D.	-	-	6073.8	689.1	3381.5	3807.5
hsa-miR-454	MIMAT0003885	64.2	70.4	67.3	4.4	N.D.	N.D.	-	-
hsa-miR-454*	MIMAT0003884	62.1	66.1	64.1	2.8	N.D.	15.1	-	-
hsa-miR-466	MIMAT0015002	67.1	86.2	76.7	13.5	35.3	N.D.	-	-
hsa-miR-483-3p	MIMAT0002173	212.1	295.1	253.6	58.6	82.5	48.4	65.4	24.1
hsa-miR-483-5p	MIMAT0004761	141.7	225.8	183.7	59.5	20.8	18.4	19.6	1.7
hsa-miR-484	MIMAT0002174	98.6	197.0	147.8	69.6	192.5	47.8	120.1	102.4
hsa-miR-485-3p	MIMAT0002176	72.6	192.6	132.6	84.8	57.1	32.5	44.8	17.4
hsa-miR-486-3p	MIMAT0004762	196.2	235.7	216.0	27.9	73.2	28.6	50.9	31.5
hsa-miR-486-5p	MIMAT0002177	119.0	145.4	132.2	18.7	734.2	258.9	496.6	336.1
hsa-miR-487b	MIMAT0003180	N.D.	46.8	-	-	12.4	21.3	16.8	6.3
hsa-miR-488*	MIMAT0002804	49.1	75.7	62.4	18.8	18.7	N.D.	-	-
hsa-miR-491-5p	MIMAT0002807	411.2	250.4	330.8	113.7	18.4	N.D.	-	-
hsa-miR-492	MIMAT0002812	77.9	153.6	115.7	53.5	13.4	N.D.	-	-
hsa-miR-493	MIMAT0003161	66.2	121.7	94.0	39.2	12.3	16.8	14.6	3.2
hsa-miR-494	MIMAT0002816	85.0	483.8	284.4	282.0	26.8	17.0	21.9	6.9
hsa-miR-497	MIMAT0002820	67.4	47.1	57.3	14.4	11.7	24.9	18.3	9.4
hsa-miR-498	MIMAT0002824	123.4	140.4	131.9	12.0	93.8	74.9	84.4	13.3
hsa-miR-502-3p	MIMAT0004775	N.D.	79.4	-	-	21.4	16.9	19.1	3.2
hsa-miR-505*	MIMAT0004776	177.5	151.3	164.4	18.5	15.9	N.D.	-	-
hsa-miR-508-3p	MIMAT0002880	N.D.	47.0	-	-	9.9	36.0	22.9	18.4
hsa-miR-508-5p	MIMAT0004778	N.D.	112.7	-	-	14.5	15.7	15.1	0.8
hsa-miR-512-5p	MIMAT0002822	N.D.	39.9	-	-	25.9	26.6	26.2	0.5
hsa-miR-513b	MIMAT0005788	N.D.	54.1	-	-	19.1	27.5	23.3	5.9
hsa-miR-514b-5p	MIMAT0015087	100.3	97.0	98.6	2.3	11.8	N.D.	-	-
hsa-miR-515-3p	MIMAT0002827	N.D.	50.0	-	-	10.7	20.0	15.3	6.5
hsa-miR-518b	MIMAT0002844	71.6	66.5	69.0	3.6	28.3	28.2	28.3	0.0
hsa-miR-518c*	MIMAT0002847	1003.4	428.1	715.7	406.8	35.2	21.0	28.1	10.0
hsa-miR-519c-5p, hsa-miR-523*, hsa-miR-518e*, hsa-miR-522*, hsa-miR-519a*, hsa-miR-519b-5p	MIMAT0002831, MIMAT0005449, MIMAT0005450, MIMAT0005451, MIMAT0005452, MIMAT0005454	N.D.	N.D.	-	-	12.9	16.2	14.6	2.3
hsa-miR-519d	MIMAT0002853	81.7	112.9	97.3	22.1	39.4	21.4	30.4	12.8
hsa-miR-519e	MIMAT0002829	48.4	202.8	125.6	109.2	19.7	25.0	22.4	3.7
hsa-miR-519e*	MIMAT0002828	44.9	53.4	49.2	6.0	N.D.	23.1	-	-
hsa-miR-521	MIMAT0002854	46.5	61.0	53.8	10.3	N.D.	14.2	-	-
hsa-miR-525-3p	MIMAT0002839	42.2	44.0	43.1	1.3	12.6	N.D.	-	-
hsa-miR-525-5p	MIMAT0002838	59.8	39.8	49.8	14.1	N.D.	N.D.	-	-
hsa-miR-526a, hsa-miR-520c-5p, hsa-miR-518d-5p	MIMAT0002845, MIMAT0005455, MIMAT0005456	81.7	51.4	66.6	21.4	11.7	N.D.	-	-
hsa-miR-526b	MIMAT0002835	97.2	71.6	84.4	18.1	11.7	23.8	17.7	8.5
hsa-miR-532-3p	MIMAT0004780	220.6	290.7	255.7	49.6	78.2	31.8	55.0	32.8
hsa-miR-532-5p	MIMAT0002888	39.9	98.1	69.0	41.2	15.8	N.D.	-	-

hsa-miR-541	MIMAT0004920	48.1	N.D.	-	-	18.4	17.4	17.9	0.7
hsa-miR-541*	MIMAT0004919	60.0	65.3	62.7	3.7	20.1	18.1	19.1	1.4
hsa-miR-542-5p	MIMAT0003340	94.4	555.7	325.1	326.2	N.D.	N.D.	-	-
hsa-miR-548a-3p	MIMAT0003251	N.D.	51.2	-	-	9.5	16.7	13.1	5.0
hsa-miR-548q	MIMAT0011163	120.2	154.8	137.5	24.5	83.4	68.4	75.9	10.6
hsa-miR-550a	MIMAT0004800	209.0	207.2	208.1	1.3	26.5	26.7	26.6	0.1
hsa-miR-550a*	MIMAT0003257	73.3	51.0	62.1	15.8	25.0	16.5	20.8	6.0
hsa-miR-550b	MIMAT0018445	119.1	98.9	109.0	14.3	40.7	28.0	34.4	9.0
hsa-miR-551a	MIMAT0003214	70.8	67.3	69.1	2.5	36.2	21.4	28.8	10.5
hsa-miR-551b	MIMAT0003233	43.4	57.1	50.2	9.7	35.2	31.4	33.3	2.7
hsa-miR-551b*	MIMAT0004794	176.8	210.6	193.7	23.9	16.0	N.D.	-	-
hsa-miR-555	MIMAT0003219	N.D.	39.0	-	-	15.0	17.9	16.5	2.1
hsa-miR-557	MIMAT0003221	595.0	451.6	523.3	101.4	300.3	264.6	282.5	25.2
hsa-miR-564	MIMAT0003228	240.9	855.2	548.0	434.3	57.8	31.7	44.8	18.5
hsa-miR-572	MIMAT0003237	50.9	43.6	47.2	5.2	16.8	N.D.	-	-
hsa-miR-574-3p	MIMAT0003239	350.6	443.9	397.3	66.0	154.1	88.1	121.1	46.7
hsa-miR-574-5p	MIMAT0004795	264.1	328.4	296.3	45.5	90.4	56.6	73.5	23.9
hsa-miR-575	MIMAT0003240	253.8	309.7	281.7	39.5	141.1	95.1	118.1	32.6
hsa-miR-576-3p	MIMAT0004796	N.D.	58.1	-	-	10.1	27.3	18.7	12.2
hsa-miR-582-5p	MIMAT0003247	N.D.	39.6	-	-	11.4	19.4	15.4	5.7
hsa-miR-583	MIMAT0003248	299.1	404.4	351.8	74.4	13.4	17.2	15.3	2.7
hsa-miR-584	MIMAT0003249	53.3	73.0	63.2	13.9	18.0	19.1	18.6	0.8
hsa-miR-589	MIMAT0004799	N.D.	N.D.	-	-	11.2	14.2	12.7	2.1
hsa-miR-589*	MIMAT0003256	48.6	45.5	47.0	2.2	11.5	18.7	15.1	5.1
hsa-miR-593	MIMAT0004802	55.5	150.1	102.8	66.9	37.0	28.4	32.7	6.0
hsa-miR-593*	MIMAT0003261	424.9	177.5	301.2	175.0	30.6	22.3	26.4	5.9
hsa-miR-595	MIMAT0003263	78.5	124.7	101.6	32.7	35.2	28.8	32.0	4.5
hsa-miR-596	MIMAT0003264	88.0	67.2	77.6	14.7	12.2	14.0	13.1	1.3
hsa-miR-598	MIMAT0003266	65.8	44.0	54.9	15.4	27.3	N.D.	-	-
hsa-miR-601	MIMAT0003269	43.7	N.D.	-	-	12.5	18.9	15.7	4.5
hsa-miR-602	MIMAT0003270	N.D.	N.D.	-	-	31.8	27.5	29.7	3.0
hsa-miR-603	MIMAT0003271	N.D.	N.D.	-	-	15.1	16.6	15.9	1.0
hsa-miR-604	MIMAT0003272	47.8	68.7	58.3	14.8	N.D.	19.0	-	-
hsa-miR-605	MIMAT0003273	43.7	58.7	51.2	10.6	32.8	22.6	27.7	7.2
hsa-miR-606	MIMAT0003274	N.D.	74.3	-	-	17.6	30.4	24.0	9.1
hsa-miR-608	MIMAT0003276	104.9	98.4	101.6	4.6	12.3	N.D.	-	-
hsa-miR-612	MIMAT0003280	65.6	163.9	114.7	69.5	25.0	19.5	22.2	3.8
hsa-miR-614	MIMAT0003282	707.5	187.7	447.6	367.6	30.1	19.9	25.0	7.2
hsa-miR-615-3p	MIMAT0003283	72.6	241.7	157.2	119.6	49.3	20.1	34.7	20.6
hsa-miR-615-5p	MIMAT0004804	339.1	343.9	341.5	3.3	337.2	264.4	300.8	51.5
hsa-miR-616	MIMAT0004805	40.3	58.4	49.4	12.8	10.0	N.D.	-	-
hsa-miR-625*	MIMAT0004808	133.4	222.5	177.9	63.0	75.7	53.4	64.6	15.7
hsa-miR-629*	MIMAT0003298	58.9	99.6	79.3	28.8	37.8	21.5	29.7	11.5
hsa-miR-631	MIMAT0003300	74.6	102.8	88.7	19.9	17.6	19.0	18.3	1.0
hsa-miR-632	MIMAT0003302	52.0	86.7	69.3	24.5	29.5	29.6	29.5	0.1
hsa-miR-634	MIMAT0003304	101.3	108.3	104.8	4.9	29.7	19.6	24.6	7.2
hsa-miR-636	MIMAT0003306	114.8	192.6	153.7	55.0	66.8	49.8	58.3	12.0
hsa-miR-637	MIMAT0003307	89.8	83.8	86.8	4.3	55.2	49.6	52.4	4.0
hsa-miR-638	MIMAT0003308	8200.9	9652.9	8926.9	1026.8	14225.4	6084.3	10154.8	5756.6
hsa-miR-642a	MIMAT0003312	85.5	192.5	139.0	75.7	65.2	36.5	50.9	20.3
hsa-miR-642b	MIMAT0018444	892.1	743.7	817.9	104.9	229.1	159.8	194.4	49.0
hsa-miR-650	MIMAT0003320	155.4	162.6	159.0	5.1	14.4	31.2	22.8	11.9
hsa-miR-654-3p	MIMAT0004814	N.D.	N.D.	-	-	55.2	22.7	39.0	23.0
hsa-miR-654-5p	MIMAT0003330	160.0	91.2	125.6	48.6	26.7	21.6	24.2	3.6
hsa-miR-657	MIMAT0003335	122.1	186.7	154.4	45.6	38.7	36.7	37.7	1.4
hsa-miR-658	MIMAT0003336	527.2	896.0	711.6	260.8	1237.0	709.2	973.1	373.3

hsa-miR-659	MIMAT0003337	146.5	193.9	170.2	33.5	13.5	34.5	24.0	14.9
hsa-miR-660	MIMAT0003338	47.5	71.7	59.6	17.1	29.2	N.D.	-	-
hsa-miR-661	MIMAT0003324	82.5	114.2	98.3	22.4	26.5	21.9	24.2	3.2
hsa-miR-663	MIMAT0003326	2578.3	3136.9	2857.6	395.0	1216.4	737.2	976.8	338.9
hsa-miR-663b	MIMAT0005867	136.8	137.6	137.2	0.6	34.7	39.9	37.3	3.7
hsa-miR-664	MIMAT0005949	38.7	144.2	91.4	74.6	30.7	18.9	24.8	8.4
hsa-miR-665	MIMAT0004952	183.0	471.8	327.4	204.2	72.0	45.9	58.9	18.5
hsa-miR-668	MIMAT0003881	137.0	208.5	172.7	50.6	75.4	38.0	56.7	26.5
hsa-miR-670	MIMAT0010357	65.7	111.1	88.4	32.1	18.6	N.D.	-	-
hsa-miR-671-3p	MIMAT0004819	160.5	247.9	204.2	61.8	29.8	18.5	24.1	8.0
hsa-miR-671-5p	MIMAT0003880	846.8	694.2	770.5	107.9	133.2	114.4	123.8	13.3
hsa-miR-675	MIMAT0004284	1622.2	487.8	1055.0	802.1	505.4	378.0	441.7	90.1
hsa-miR-675*	MIMAT0006790	195.1	200.6	197.8	3.9	89.4	72.2	80.8	12.2
hsa-miR-676	MIMAT0018204	43.8	46.9	45.4	2.2	18.0	24.9	21.4	4.9
hsa-miR-711	MIMAT0012734	2446.0	3546.0	2996.0	777.9	444.9	190.3	317.6	180.0
hsa-miR-718	MIMAT0012735	228.1	622.1	425.1	278.6	178.1	124.6	151.3	37.8
hsa-miR-7-2*	MIMAT0004554	N.D.	49.8	-	-	17.8	17.5	17.6	0.2
hsa-miR-720	MIMAT0005954	3950.5	10204.6	7077.6	4422.3	262.3	162.3	212.3	70.7
hsa-miR-744	MIMAT0004945	517.7	1208.5	863.1	488.5	549.0	392.0	470.5	111.0
hsa-miR-744*	MIMAT0004946	N.D.	66.9	-	-	13.0	15.6	14.3	1.8
hsa-miR-760	MIMAT0004957	482.8	523.2	503.0	28.6	171.7	79.2	125.4	65.4
hsa-miR-761	MIMAT0010364	54.1	94.7	74.4	28.7	14.9	N.D.	-	-
hsa-miR-762	MIMAT0010313	13612.1	22883.6	18247.9	6555.9	14567.0	9750.5	12158.7	3405.8
hsa-miR-764	MIMAT0010367	79.9	143.5	111.7	45.0	34.7	15.4	25.1	13.6
hsa-miR-765	MIMAT0003945	55.2	67.8	61.5	8.9	12.7	16.1	14.4	2.3
hsa-miR-766	MIMAT0003888	300.4	246.8	273.6	37.9	104.5	60.9	82.7	30.8
hsa-miR-767-3p	MIMAT0003883	52.9	105.9	79.4	37.5	19.2	13.9	16.6	3.7
hsa-miR-769-3p	MIMAT0003887	39.1	77.6	58.3	27.2	42.1	31.9	37.0	7.2
hsa-miR-770-5p	MIMAT0003948	N.D.	61.8	-	-	18.7	15.9	17.3	2.0
hsa-miR-874	MIMAT0004911	267.9	175.1	221.5	65.7	33.5	32.4	32.9	0.8
hsa-miR-875-3p	MIMAT0004923	40.0	48.7	44.4	6.2	13.6	N.D.	-	-
hsa-miR-877	MIMAT0004949	181.0	112.3	146.7	48.6	26.1	14.1	20.1	8.5
hsa-miR-877*	MIMAT0004950	228.7	447.3	338.0	154.6	89.0	59.7	74.4	20.7
hsa-miR-885-3p	MIMAT0004948	101.7	174.3	138.0	51.3	27.7	N.D.	-	-
hsa-miR-885-5p	MIMAT0004947	134.2	234.6	184.4	71.0	56.8	30.3	43.5	18.8
hsa-miR-887	MIMAT0004951	127.2	137.3	132.2	7.2	578.1	284.0	431.1	208.0
hsa-miR-888	MIMAT0004916	N.D.	58.0	-	-	13.1	16.5	14.8	2.4
hsa-miR-888*	MIMAT0004917	104.5	46.6	75.5	40.9	N.D.	N.D.	-	-
hsa-miR-891a	MIMAT0004902	61.9	75.5	68.7	9.6	17.8	19.0	18.4	0.9
hsa-miR-892a	MIMAT0004907	N.D.	46.9	-	-	16.5	24.3	20.4	5.5
hsa-miR-892b	MIMAT0004918	63.6	103.9	83.7	28.5	28.9	28.9	28.9	0.0
hsa-miR-920	MIMAT0004970	985.3	300.3	642.8	484.3	54.8	26.8	40.8	19.8
hsa-miR-92a	MIMAT0000092	96.2	362.0	229.1	188.0	543.5	132.1	337.8	290.9
hsa-miR-92a-2*	MIMAT0004508	556.9	2385.0	1470.9	1292.7	1458.7	825.7	1142.2	447.6
hsa-miR-92b	MIMAT0003218	86.5	242.9	164.7	110.6	217.6	48.4	133.0	119.7
hsa-miR-92b*	MIMAT0004792	1688.9	1783.3	1736.1	66.7	713.3	349.8	531.6	257.1
hsa-miR-93	MIMAT0000093	N.D.	291.1	-	-	487.2	78.7	282.9	288.8
hsa-miR-934	MIMAT0004977	112.7	206.9	159.8	66.6	N.D.	N.D.	-	-
hsa-miR-935	MIMAT0004978	53.5	88.5	71.0	24.7	46.7	23.3	35.0	16.6
hsa-miR-936	MIMAT0004979	91.9	118.9	105.4	19.1	12.9	17.1	15.0	2.9
hsa-miR-937	MIMAT0004980	54.5	157.0	105.7	72.5	45.5	28.3	36.9	12.2
hsa-miR-938	MIMAT0004981	N.D.	N.D.	-	-	15.8	14.4	15.1	1.0
hsa-miR-939	MIMAT0004982	651.3	437.0	544.1	151.5	136.3	68.3	102.3	48.1
hsa-miR-940	MIMAT0004983	194.5	264.9	229.7	49.8	86.4	66.4	76.4	14.2
hsa-miR-943	MIMAT0004986	64.0	86.8	75.4	16.1	47.0	30.3	38.7	11.8
hsa-miR-99a	MIMAT0000097	43.0	273.9	158.5	163.2	13.7	N.D.	-	-

hsa-miR-99b	MIMAT0000689	60.7	133.5	97.1	51.5	33.3	19.2	26.3	9.9
hsa-miR-99b*	MIMAT0004678	56.5	71.5	64.0	10.6	24.8	14.6	19.7	7.2

N.D.: Not detected
